# Enhancing nutritional niche and host defenses by modifying the gut microbiome

**DOI:** 10.15252/msb.20209933

**Published:** 2022-11-15

**Authors:** Qing Sun, Nic M Vega, Bernardo Cervantes, Christopher P Mancuso, Ning Mao, Megan N Taylor, James J Collins, Ahmad S Khalil, Jeff Gore, Timothy K Lu

**Affiliations:** ^1^ Synthetic Biology Center MIT Cambridge MA USA; ^2^ Department of Chemical Engineering Texas A&M University College Station TX USA; ^3^ Department of Physics MIT Cambridge MA USA; ^4^ Biology Department Emory University Atlanta GA USA; ^5^ Institute for Medical Engineering & Science and Department of Biological Engineering MIT Cambridge MA USA; ^6^ Broad Institute of MIT and Harvard Cambridge MA USA; ^7^ Microbiology Graduate Program MIT Cambridge MA USA; ^8^ Biological Design Center Boston University Boston MA USA; ^9^ Department of Biomedical Engineering Boston University Boston MA USA; ^10^ Wyss Institute for Biologically Inspired Engineering Harvard University Boston MA USA; ^11^ Department of Electrical Engineering and Computer Science MIT Cambridge MA USA; ^12^ Department of Biological Engineering MIT Cambridge MA USA

**Keywords:** bacteria community, cellulose, gut microbiome, nutrition, pathogen, Biotechnology & Synthetic Biology, Microbiology, Virology & Host Pathogen Interaction

## Abstract

The gut microbiome is essential for processing complex food compounds and synthesizing nutrients that the host cannot digest or produce, respectively. New model systems are needed to study how the metabolic capacity provided by the gut microbiome impacts the nutritional status of the host, and to explore possibilities for altering host metabolic capacity via the microbiome. Here, we colonized the nematode *Caenorhabditis elegans* gut with cellulolytic bacteria that enabled *C. elegans* to utilize cellulose, an otherwise indigestible substrate, as a carbon source. Cellulolytic bacteria as a community component in the worm gut can also support additional bacterial species with specialized roles, which we demonstrate by using *Lactobacillus plantarum* to protect *C. elegans* against *Salmonella enterica* infection. This work shows that engineered microbiome communities can be used to endow host organisms with novel functions, such as the ability to utilize alternate nutrient sources or to better fight pathogenic bacteria.

## Graphical abstract

A microbe‐host interaction model is developed by colonizing *C. elegans* with functional bacteria that allow digesting long‐chain cellulose. Direct benefits include increased host larval yield and protection of other gut species against pathogens.

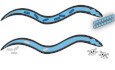

## Introduction

The gut microbiome is a complex community of microbes, the ecology of which is intimately tied to the physiology of its host organism (Sampson & Mazmanian, 2015; Barratt *et al*, 2017). These symbiotic microbes have been shown to contribute to the nutritional status and metabolism of the host (Shreiner *et al*, 2015). Understanding how various species of microorganisms constituting the microbiome affect their host is fundamental to deriving potential benefits from manipulating the microbiome.

Gut microbe metabolism affects the health of animal hosts in numerous ways, from the liberation of indigestible nutrients (Tremaroli & Bäckhed, 2012) to the modification of host‐secreted bile acids (Rowland *et al*, 2018), to the synthesis of certain vitamins and neurotransmitters at significant levels (Sampson & Mazmanian, 2015). Moreover, metabolic interactions among members of the microbiota help maintain community structure, thereby, preventing pathogen invasion or other dysbioses (Shreiner *et al*, 2015).

Carbohydrates are important sources of energy for both microbes and animals (Tremaroli & Bäckhed, 2012). Simple sugars such as glucose and lactose can be easily absorbed by hosts, but more complex carbohydrates and plant polysaccharides, including cellulose, xylans, resistant starch, and inulin, are not as easily digested. The gut microbiome can confer caloric benefits upon its host by breaking down these ingested plant carbohydrates, which the host enzymes cannot digest (Rowland *et al*, 2018), as has been demonstrated for termites (Bourguignon *et al*, 2018) and ruminants (Clemmons *et al*, 2019). The gut microbiome can also evolve to adapt to new carbohydrate sources when hosts change their eating habits. For example, the Japanese, as a result of their diet, have intestinal microbiomes that have acquired enzymes for processing the algal carbohydrates in seaweed through horizontal gene transfer from marine bacteria (Hehemann *et al*, 2012). However, research into the use of non‐native bacteria to perform nutritional functions for other animal hosts has been limited.

In this study, we engineered a functional microbiome in a simple animal gut to allow host utilization of a complex carbohydrate source. We used the nematode *Caenorhabditis elegans* as the animal host as it has been extensively used as a model system to elucidate mechanisms of interaction between prokaryotes and their hosts (Jones & Ashrafi, 2009; Zhang *et al*, 2017). Specifically, we colonized *C. elegans* with cellulolytic bacteria that break down cellulose in the gut, such that the released glucose is available as a nutrient for both *C. elegans* itself and the colonizing bacteria. To move this system toward higher community complexity, as occurs in a natural gut microbiome, we added an additional bacterial species with the specialized function of preventing infection by pathogens. Our work indicates that by assembling a functional community in the *C. elegans* gut, we can extend the nutrition processing and pathogen inhibition capacities of the animal host.

## Results

### Cellulolytic bacterial colonization and function in *C. elegans* gut

**Figure 1:**
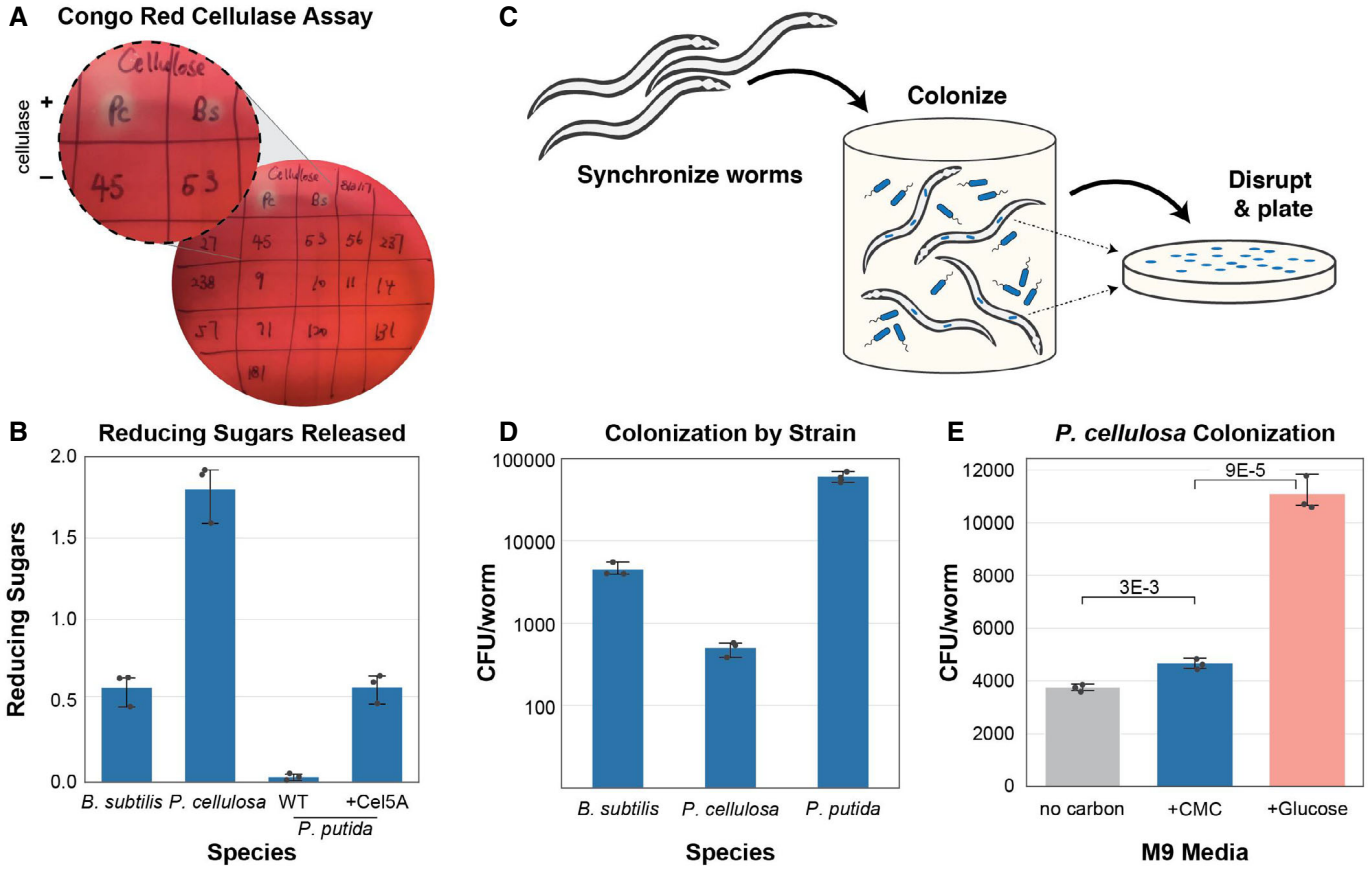
Bacterial colonization and function in *C. elegans* gut Cellulase functional screening by Congo Red assay. Halos on the plate indicate cellulase activity. Pc: *Pseudomonas cellulosa*, Bs: *Bacillus subtilis*. Numbered strains are from the *C. elegans* native gut microbiome as listed in Appendix Table S1.Production of reducing sugars by cellulose‐degrading bacterial strains.Schematic of bacterial colonization and analysis procedure. Synchronized L1 wild‐type worms were grown on selected strains of live bacteria and then disrupted for colonization analysis by plating and colony counting.Colony‐forming units (CFU) of various bacterial species in the worm gut.
*Caenorhabditis elegans* colonized with cellulolytic *P. cellulosa* and incubated with different carbon sources. Data information: All the experiments were performed in biological triplicates. Student's *t*‐test was used for statistical analysis. Error bars represent 95% confidence intervals of the mean.

To introduce a novel carbon processing capability to *C. elegans*, we started by selecting cellulose‐degrading microbes as potential intestinal colonists. To identify these functional bacteria members, we assembled a collection of bacteria native to the *C. elegans* gut (Dirksen *et al*, 2016; see Appendix Table S1 for a full list) and tested these species for their ability to utilize carboxymethyl cellulose (CMC) and other carbon sources by measuring bacterial optical density following *in vitro* incubation (Appendix Fig S1). Additionally, we checked the cellulose degradation capacity for each bacterial strain using a Congo Red assay (Teather & Wood, 1982; Houfani *et al*, 2017). As Congo Red stains glucose polymers, a halo surrounding the colony indicates cellulase activity (Fig 1A). Although the investigation of the functional capacity of the *C. elegans* native microbiome indicates that some rare members of this microbiome can degrade cellulose (Zimmermann *et al*, 2020), none of the native *C. elegans* microbiome isolates in this particular set of strains (Fig 1A) exhibited detectable cellulase activity, which reflects their lack of cellulose processing capability. To further identify cellulolytic bacteria, we screened a set of soil bacteria (Appendix Table S1) and identified *Pseudomonas cellulosa* and *Bacillus subtilis* as cellulase‐active bacteria (Fig 1A). Of these strains, *P. cellulosa* produced the highest levels of reducing sugars, including glucose (Fig 1B), suggesting that this strain could potentially be useful as a nutritional endosymbiont for the *C. elegans* host, supplying nutrients that would otherwise be unavailable. After screening, we also kept one of the non‐cellulolytic bacterial strains, *Pseudomonas putida*, as a negative control and for use in future engineering.

We next sought to determine whether a cellulolytic organism could colonize the worm intestine. *C. elegans* feed on bacteria, a certain percentage of which escape digestion and colonize the gut (Schulenburg & Félix, 2017; Vega & Gore, 2017). In these experiments, synchronized adult N2 worms were used for colonization to provide a genetically homogeneous population of mature hosts. We allowed the worms to be colonized by culturing them in liquid bacterial culture to ensure that all the worms experienced a uniform environment (Vega & Gore, 2017). Worms were then collected, washed, and mechanically disrupted to release the intestinal bacteria. These suspensions were then plated on agar to estimate the extent of bacterial colonization (Fig 1C). Naturally cellulolytic bacteria, *P. cellulosa* and *B. subtilis*, as well as the natively non‐cellulolytic bacterium, *Pseudomonas putida*, which had been engineered to produce endoglucanase (see Materials and Methods), each achieved a colonization density of ~10^2^–10^4^ colony‐forming units (CFU) per worm (Fig 1D). Colonization was stable for at least 2 days in the *C. elegans* gut. All of these bacterial species were therefore able to colonize the host intestine, albeit at different population sizes, at colonization levels comparable to those of native *C. elegans* gut strains (Appendix Figs S2 and S3).

To determine whether bacteria identified as being cellulolytic *in vitro* could hydrolyze complex sugars in the gut environment, we pre‐colonized *C. elegans* with the cellulolytic strain *P. cellulosa*. This strain was chosen for its strong ability to degrade CMC and release reducing sugars, including glucose (Fig 1B and Appendix Fig S4). Since *P. cellulosa* grows to low densities in culture, we used *Escherichia coli* OP50 as a supplemental food source to ensure that worms had adequate nutrition during growth. We used OP50 because *C. elegans* is usually grown monoxenically in the laboratory using *E. coli* strain OP50 as a food source (Brenner, 1974). In these experiments, synchronized adult N2 worms were grown on solid plates instead of liquid media to allow efficient feeding on these bacteria and therefore increase the rate of colonization. As we intended to use fecundity as one measure of carbon uptake in these experiments and as *C. elegans* fecundity shows a strong response to starvation (adult reproductive diapause) when food is withdrawn in the mid‐ to late L4 stage, feeding and colonization on live bacteria were stopped at 46 h post‐L1 on plates (Seidel & Kimble, 2011). Worms raised on mixed lawns of *P. cellulosa* and *E. coli* OP50 showed intestinal colonization by *P. cellulosa* (Fig 1D), indicating that this bacterial species is able to colonize the host. Pre‐colonized worms were then incubated for 24 h in liquid media (M9 worm buffer) with: (i) no carbon source, (ii) cellulose (in the form of CMC), or (iii) glucose. After incubation, worms were washed and digested to check gut bacterial density. We found that M9 buffer with CMC as a carbon source enhanced the colonization density of *P. cellulosa* compared with no carbon source (Fig 1E). This experiment demonstrated that a cellulolytic bacterium, *P. cellulosa*, could utilize CMC as a carbon source in the *C. elegans* gut. Glucose as a carbon source further enhanced colonization density; this result was not surprising because free sugar monomers are an easier‐to‐access carbon source than CMC, which can be utilized only after it has been hydrolyzed to glucose.

### Intestinal cellulolytic bacteria allow host utilization of cellulose

Next, we sought to determine whether colonization by cellulolytic bacteria would allow the host to use the otherwise indigestible cellulose carbon source. *C. elegans* is able to use simple carbohydrates, including glucose (Lu & Goetsch, 1993), but does not produce enzymes that degrade cellulose. We hypothesized that if cellulolytic organisms in the gut could liberate carbon in a form that is available and metabolizable by the host, colonization of the gut with a cellulolytic microbiome would provide this host with the ability to use cellulose as a growth substrate. Again, we chose *P. cellulosa* as the colonizing bacteria. Three approaches, described below, were used to investigate whether *C. elegans* utilized glucose released by gut bacteria.

**Figure 2:**
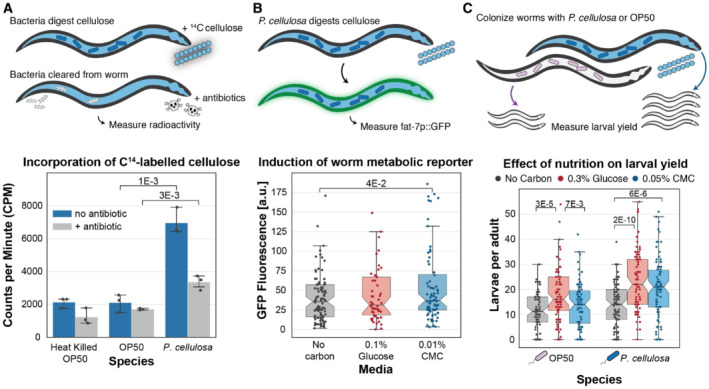
Intestinal cellulolytic bacteria allow host utilization of cellulose Incorporation of ^14^C‐labeled cellulose. Worms were first colonized with cellulolytic bacteria and then incubated with ^14^C‐cellulose to allow carbon utilization. Antibiotic treatment was applied before ^14^C measurement of worms to eliminate bacterial interference in isotopic reading. ^14^C‐cellulose incorporation measurement reflected the isotopic carbon incorporation in *C. elegans*.Induction of the worm *fat‐7p::GFP* metabolic reporter. Adult worms pre‐colonized with *P. cellulosa* were incubated in liquid media for 24 h with the indicated carbon sources, and GFP fluorescence was read in individual worms on a BioSorter large object sorter.Effect of nutrition on larval yield. Pre‐colonized worms were separated into the individual wells of a 384‐well plate, and larvae counts in each well were read after 48 h. Larval yield comparison indicates the nutritional benefit of cellulolytic activity. Data information: All the experiments were performed in biological triplicates. Student's *t*‐test was used for statistical analysis. Student's *t*‐test was used for statistical analysis. In (A) experiments were performed in biological triplicates using 200 worms per data point. In (A and B) error bars represent 95% confidence intervals for the mean. In (B) each point represents a different worm in biological replicates, *n* = 96, *n* = 53, and *n* = 80 from left to right. In (C) the central band represents the median, notches represent 95% confidence intervals for the median, the box edges represent the inter quartile range (IQR) and the whiskers extend an additional 1.5 IQR beyond the boxes. In (C) each point represents a different worm in biological replicates, *n* = 74, *n* = 79, *n* = 79, *n* = 89, *n* = 88, *n* = 86 from left to right.

First, we used radionucleotide (^14^C) incorporation as a direct measurement of cellulose‐derived carbon incorporation into worm biomass. Briefly, adult wild‐type worms were colonized with *E. coli* OP50 (non‐cellulose degrader) or *P. cellulosa*, then incubated in S medium with heat‐killed *E. coli* OP50 and trace ^14^C labeled CMC (radiolabeled cellulose purified from *Arabidopsis thaliana*; Faik *et al*, 2002) for 24 h to allow bacterial degradation of the substrate to proceed. After incubation on the radionucleotide substrate, worms were washed and treated with an antibiotic cocktail for 24 h to remove bacteria, in order to eliminate the effects of bacterial biomass on scintillation counts and observe the incorporation of cellulose‐derived carbon into the germ‐free worm biomass. A high‐throughput antibiotic susceptibility screen of the bacterial strains (including bacteria native to the *C. elegans* gut and the soil bacteria listed in Appendix Table S1) was conducted to determine an appropriate antibiotic cocktail to eliminate these species from the *C. elegans* gut (Appendix Fig S5). Clearance by the antibiotic cocktail was tested by imaging worms fed with fluorescently labeled *Pseudomonas putida* (Appendix Fig S6). After clearing the bacterial biomass, worms were mechanically digested in batches (200 worms per sample), and ^14^C incorporation was measured using scintillation counts. ^14^C incorporation was significantly higher for *P. cellulosa* colonized worms than for *E. coli* colonized worms, before and after clearance with antibiotics (Fig 2A), demonstrating that cellulolytic bacteria in the *C. elegans* gut had hydrolyzed ^14^C labeled CMC and that the hydrolyzed glucose had been taken up by the host, as well as by the microbes.

Second, we confirmed that incorporation of carbon from microbiome‐digested CMC affected host metabolism by using a fluorescent reporter of host nutritional status (Lee *et al*, 2015). We first validated that *fat‐7p*::GFP, which is expressed in the intestinal cells of *C. elegans* (Nomura *et al*, 2010; Ma *et al*, 2015), was a reliable reporter for carbon uptake by the host, and determined an optimal glucose concentration of 0.1–0.2% (w/v) for these assays (Appendix Fig S7). Young adult wild‐type worms (N2 strain, 46 h after L1 instar) were transferred from plates of live *E. coli* OP50 + *P. cellulosa*, where they had been colonized during growth by live bacteria, to S medium with heat‐killed *E. coli* (to provide nitrogen and other nutrients) ± glucose or CMC. After we washed the worms with 0.1% Triton X‐100 to remove most external bacteria (Vega & Gore, 2017), gentamicin (10 μg/ml) was used to prevent the growth of bacteria outside the worms, ensuring that nutritional benefits would be conferred by resident bacteria in the gut. When provided with CMC as a carbon source, worms pre‐colonized with *P. cellulosa* showed an increase in fluorescence from the reporter *fat‐7p*::GFP (Fig 2B), indicating an increase in nutrients available to the host.

Third, we measured the benefits of carbon incorporation into the new host biomass via larval output, to determine whether colonization by cellulose‐degrading bacteria could improve the reproductive fitness of the host. Larval output in the worm is a function of nutritional status and can be initiated by adding nutrients after starvation at a specific point in the young adult‐to‐mature adult transition (Seidel & Kimble, 2011). We, therefore, transferred worms from solid media colonization plates to liquid culture supplemented with glucose or CMC 46 h after plating L1 larvae, to capture worms at the vulnerable point in this transition. Here, worms were placed into individual wells of a 384‐well plate, to allow enumeration of larval yield for single adults. After 48 h in liquid media with a carbon source, the number of larvae produced by individual worms was counted. Worms colonized with *P. cellulosa*, but not with *E. coli* OP50 alone, produced more larvae per adult worm when CMC was provided in the medium (Fig 2C and Appendix Fig S6), again indicating that colonization by the cellulolytic bacterial species allows the worms to benefit from CMC as a carbon source made available by these resident bacteria.

### Bacteria community protects *C. elegans* against pathogen invasion of the gut

**Figure 3:**
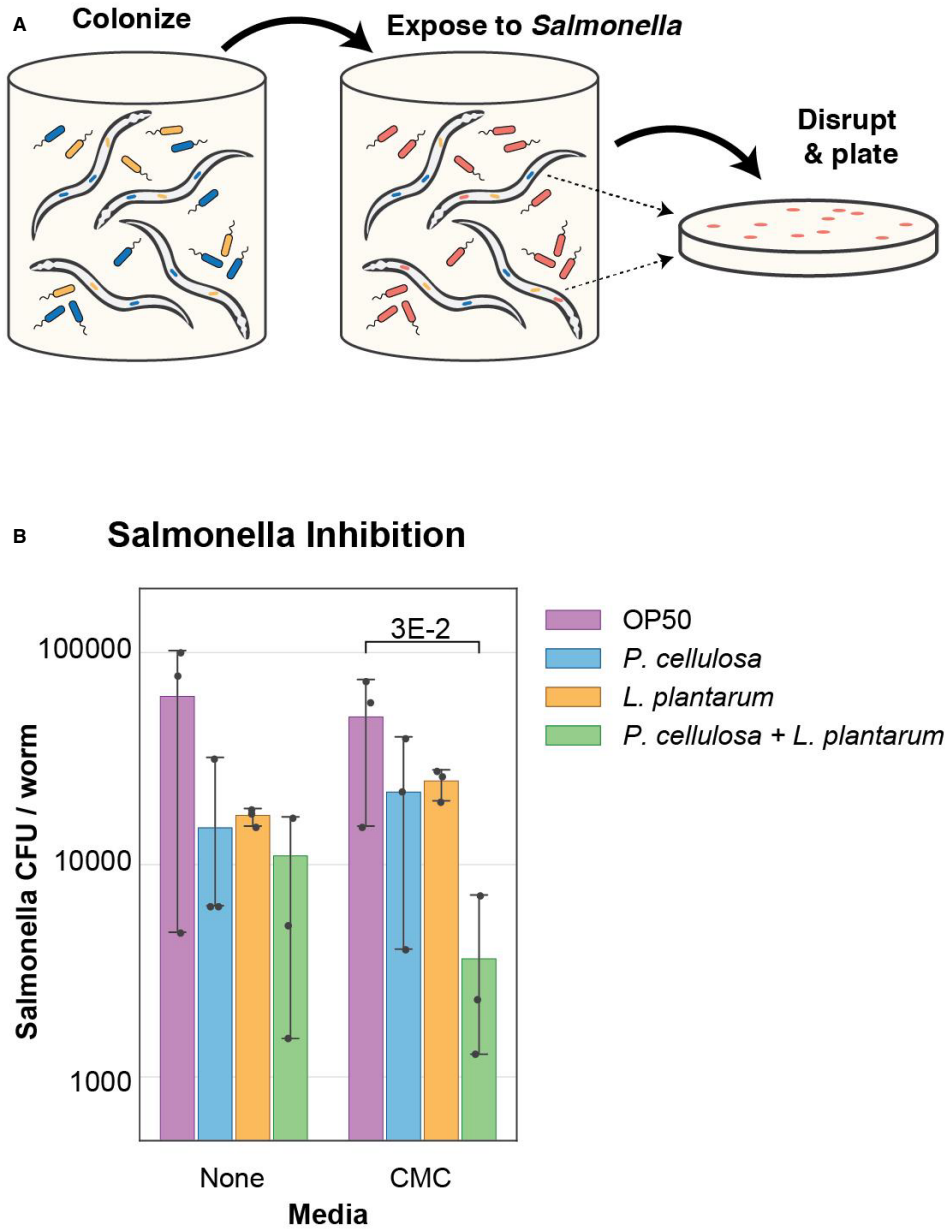
Addition of probiotic *L. plantarum* (Lp) protects *C. elegans* against invasion of the gut by *Salmonella enterica* (pathogen) Synchronized adult worms were colonized for 2 days on plates containing *E. coli* OP50 + *P. cellulosa* (Pc), *E. coli* OP50 + Lp, or *E. coli* OP50 + Pc + Lp. A 1 h exposure to *S. enterica* in liquid culture was used to colonize worms with this pathogen.After 36 h outgrowth of the pathogen, worms pre‐colonized with *P. cellulosa* in the presence of *L. plantarum* (*E. coli* OP50 + Pc + Lp) showed lower pathogen burden than *P. cellulosa* monocolonized worms when CMC was used as the carbon source. Data shown are fold change *Salmonella* infection (CFU/worm) relative to the *P. cellulosa*‐only condition. Error bars represent 95% confidence interval of the mean. CMC = 0.01% carboxymethylcellulose (w/v). All experiments were performed in biological triplicates. Student's *t*‐test was used for the statistical analysis.

Alongside these efforts, we sought to determine whether adding a co‐colonizing microbial species would produce a community with additional functionality for the host. As an alternative to adding an additional cellulose‐degrading bacterium, we sought to take advantage of the probiotic properties of *Lactobacillus plantarum*, as lactic acid bacteria have previously been shown to have probiotic effects in their hosts, including protection against pathogens (Ikeda *et al*, 2007; Bucková *et al*, 2018; Mao *et al*, 2018). We hypothesized that adding this probiotic to the gut community could prevent disruption of the community by an intestinal pathogen (here, *Salmonella enterica* LT2). To determine the effect of *L. plantarum* on pathogen colonization, worms were pre‐colonized with *P. cellulosa* on solid media as previously described, with and without the addition of *L. plantarum* (10^9^ CFU/plate). After colonization, worms were exposed to *S. enterica* LT2 (10^8^ CFU/ml) for 1 h in liquid culture (Fig 3A). Following this initial exposure, worms were washed to remove external pathogens and incubated for 36 h to allow infection to progress (Fig 3B). We found that *P. cellulosa* and *L. plantarum*, alone or in combination, modestly suppressed pathogen proliferation relative to the control condition (colonization by *E. coli* OP50), but there was no significant difference between the *P. cellulosa* only and the *P. cellulosa* and *L. plantarum* combination conditions without a carbon source (Fig 3B).

In the presence of CMC as carbon source, however, *P. cellulosa* and *L. plantarum* in combination suppressed the proliferation of *Salmonella* by 10‐fold compared with what was seen in worms colonized by either one of these strains alone (Fig 3B and Appendix Fig S8). These data suggest that the progression of infection in *C. elegans* can be affected by the resident microbiome. Moreover, the presence of CMC as a carbon source enhanced the community's capability to suppress pathogens, demonstrating that provision of a specific substrate for the cellulose degrader improved overall community performance. Our hypothesis is that simple sugars released by *P. cellulosa* promote *L. plantarum*'s capability to fight against the pathogen. Although there have been efforts using single‐species probiotics to fight against pathogen colonization (Mao *et al*, 2018; Kissoyan *et al*, 2019), our work shows that a simple microbial community can suppress pathogens in the *C. elegans* intestine. The strains in a community, compared with the single‐strain system, appear to benefit from the complex mix of available nutrients and acquire an enhanced ability to suppress organisms that are pathogenic to the host.

## Discussion

**Figure 4:**
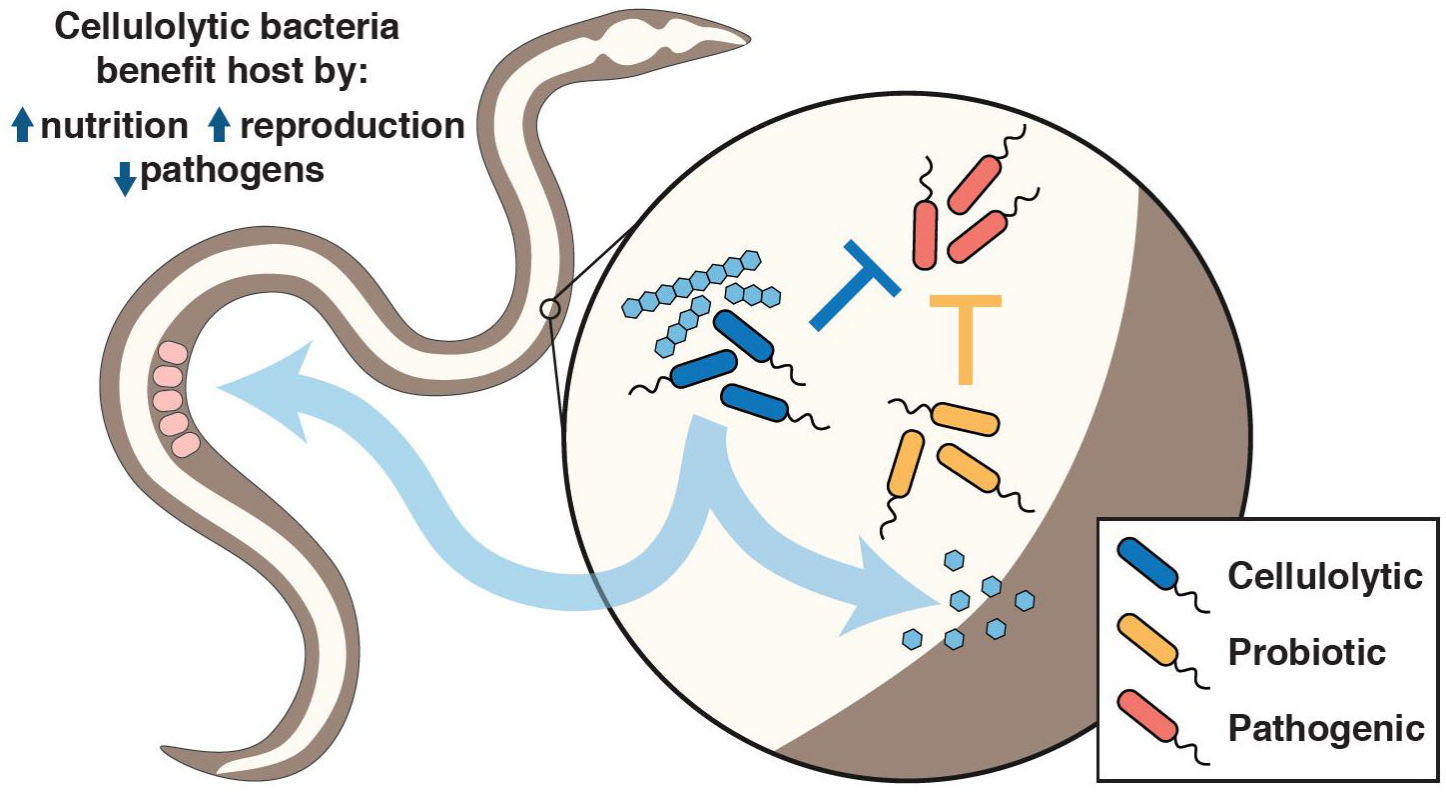
Benefits provided by colonization of the *C. elegans* gut by a heterologous microbial community Cellulolytic bacteria are able to break down cellulose, such that the released glucose can serve as nutrition for both *C. elegans* and colonized bacteria. Additional bacterial species can improve resistance against pathogenic bacteria.

In this study, we developed a model of metabolic microbe–host interactions and directly demonstrated that colonization with heterologous bacteria enables *C. elegans* to digest and incorporate carbon from previously indigestible long‐chain carbohydrates. In addition to isotopic measurement of carbon incorporation, we demonstrate direct benefits both to the host, with increased larval yield, and to other gut species, which in turn may provide protective effects against pathogens (Fig 4).

We developed assays to quantify the effects of an exogenously introduced microbiome on the nutritional status of *C. elegans*. Datasets collected toward this end include high‐throughput *in vitro* carbon source growth screens to identify bacteria capable of degrading complex carbon sources, and *in vitro* and *in vivo* screens to identify antibiotic cocktails for the removal of colonized bacteria from the *C. elegans* gut. These assays can support the use of these organisms as a convenient model for future studies of microbiota–host interactions.

The simple systems described here have yielded insights about the intricacies of inter‐species interactions in the potential pitfalls of designing a novel microbiome. In our experiments, the use of a facultatively interacting taxon (*L. plantarum*) allowed us to add a new functional capacity (resilience against pathogen invasion) by capitalizing on the particular properties of this facultative species. This new function of the community was boosted when we exposed it to CMC, which can be utilized by cellulolytic *P. cellulosa* in the community, demonstrating that distinct bacteria can interact to combat pathogens via cellulose utilization. These results, consistent with other research (Miller *et al*, 2018) in this area, indicate that understanding interactions between bacterial strains within an *in vivo* host environment will be critically important in selecting novel colonists for a host microbial ecosystem. Community assembly starting from modular orthogonal interactions offers a promising approach for designing microbiome communities (Chu & Adler, 2015; Friedman *et al*, 2017; Kong *et al*, 2018; Medlock *et al*, 2018; Venturelli *et al*, 2018; Lopez *et al*, 2020). Our results are also consistent with current thought on the use of “prebiotic” nutritional adjuvants to support the engraftment and function of specific beneficial microbes in the gut. Recent studies that utilize porphyran as an orthogonal metabolite to enable strain engraftment (Kearney *et al*, 2018; Shepherd *et al*, 2018) are likewise consistent with this view.

Engineering the gut microbiome could permit hosts to digest new food sources or enhance the efficiency of current nutrition processing capabilities. With rapid progress in enzyme evolution (Nielsen & Keasling, 2016) and microbiome engineering (Johns *et al*, 2016) techniques, facilitated by developments with model host organisms, engineering the gut microbiome could accelerate nutrient processing in livestock or expand diets to include entirely new food sources.

## Materials and Methods

### Bacterial species, worm strains, and culturing

Bacterial species used in this study are listed in Appendix Table S1, along with species‐specific growth media. In absence of specific culture requirements, bacterial cultures were routinely inoculated from glycerol stock and grown in LB media in a 30 or 37°C shaking incubator or a benchtop shaker (23°C) for 24–48 h before use.

Unless otherwise stated, N2 wild‐type *C. elegans* (Caenorhabditis Genetic Center) were used in these experiments. Cultivation of all worms used here was performed at 25°C on NGM plates seeded with *E. coli* OP50. Synchronization of worms for these experiments was performed using standard protocols (Stiernagle, 2006); briefly, worms were cultivated on NGM to obtain large numbers of gravid adults hermaphrodites, and eggs were isolated from these plates via bleach‐NaOH lysis of adults. Eggs were rinsed 5–6× in M9 worm buffer to remove the hypochlorite solution and allowed to hatch out in 5 ml of M9 worm buffer overnight, after which the synchronized and starved L1s could be used for experiments. When reproductively sterile adult worms were desired, L1 larvae were plated onto NGM + 1 mM IPTG + 50 μg/ml plates seeded with *E. coli* expressing *pos‐1* RNAi from a plasmid (Rual *et al*, 2004); worms grown to adulthood on these plates produce inviable eggs and are therefore reproductively sterile.

### Cellulolytic bacteria screening

A Congo Red assay was used to determine cellulose hydrolyzing capability of bacteria. 1% CMC plates seeded with bacteria were cultured overnight at 30°C. The plates were then flooded with 0.1% Congo Red for 15–20 min and subsequently rinsed with 1 M NaCl solution. A halo formed around the colony indicates cellulase indicates cellulase activity. For resting cell assays, cells in liquid culture were first washed once with buffer containing 50 mM Tris–HCl (pH 8.0) and suspended in 1% CMC (carboxymethyl cellulose) from Sigma. Samples were collected after 1 h and immediately mixed with 0.5 ml of DNS reagents (10 g/l dinitrosalicylic acid, 10 g/l sodium hydroxide, 2 g/l phenol, 0.5 g/l sodium sulfite). After incubation at 95°C for 10 min, 1 ml of 40% Rochelle salts was added to the color before measuring the absorbance at 575 nm (Sun & Chen, 2016).

### Bacteria colonization and analysis

Washed adult synchronized worms were resuspended in S medium and moved in 500 μl aliquots to 15 ml culture tubes with a final concentration of ~1,000 worms/ml. In total, 500 μl of bacterial suspension at 2× desired concentration was added to each culture tubes. Culture tubes were incubated with shaking at 300 rpm at 25°C. After 24 h, colonized worms were washed 3× with M9 worm buffer +0.1% Triton X‐100 and 2× with M9 worm buffer to remove external bacteria. Worms were subsequently chilled to 4°C for 15 min to stop peristalsis and treated for 10 min with a 1:2,000 solution of commercial bleach to remove any remaining external bacteria, then washed 2× in M9 worm buffer +0.1% Triton X‐100 to remove bleach prior to disruption.

For manual disruption, worms were transferred to 3 ml M9 worm buffer +1% Triton X‐100 in a small (35 cm) petri dish (Fisher Scientific). Individual worms were pipetted out and transferred to 0.5 ml clear microtubes (Kimble Kontes) for manual disruption with a motorized pestle (Kimble Kontes Pellet Pestle with blue disposable pestle tips, Fisher Scientific, all digestions in 20 μl of buffer). Magnification was provided by a magnifying visor (Magni‐Focuser Hands Free Binocular Magnifier 3.5×). After disruption, tubes were centrifuged 2 min at 16,099 *g* to collect all material, and the resulting pellet was resuspended in 180 μl M9 worm buffer (final volume 200 μl) before transfer to 96‐well plates for serial dilution in 1× phospho‐buffered saline (PBS). Ten‐, 100‐ and 1,000‐fold dilution samples were plated on LB or ATCC Medium 2720 plates to check the colonization density.

### Colonized bacteria benefit from CMC

Synchronized L1 worms from the N2 wild‐type lineage were plated on solid agar on mixed lawns of *E. coli* OP50 and *P. cellulosa*, and allowed to grow to adulthood. Following this colonization, day 0 adult worms were separated into two aliquots. One went through disruption for colonization density as described above. The second aliquot was divided between wells of a 24‐well plate with S medium, S medium with 0.1% glucose and S medium with 0.1% CMC (final volume 1 ml/well), covered with a BreatheEasy gas‐permeable membrane, and incubated with shaking at 200 rpm at 25°C for 24 h. Individual samples went through disruption and plating to check on nutritional effects on colonization density.

### Measurement of ^14^C‐carbon incorporation

Each gut colonization type was done in triplicate using 200 N2 synchronized worms. Samples were incubated at room temperature, with gentle mixing, in 1 ml M9 Worm buffer containing 0.5% Carboxy‐methyl cellulose and 1 μCi/ml ^14^C‐Uniformly labeled cellulose from *A. thaliana* for 72 h. Antibiotic cocktails (100 μg/ml ciprofloxacin and 500 μg/ml carbenicillin) were provided at 48 h. Scintillation counts were performed after washing the worms with M9 buffer containing 0.1% TritonX. Washing of the worms was repeated until the scintillation counts in the wash supernatant reached background levels. Scintillation counts were performed using liquid scintillation analyzer (PerkinElmer Tri‐Carb 2910 TR) in a high‐efficiency LSC‐cocktail (PerkinElmer Ultima Gold).

### Antibiotics cocktails for gut bacteria elimination

A high‐throughput antibiotic susceptibility screen over our collection of soil bacteria was conducted with incubating bacteria antibiotics pooled and checked on bacteria density afterward. Once an effective antibiotic cocktail was identified, the best conditions to kill bacteria in the worm gut were determined. Worms were colonized with YFP‐labeled *Pseudomonas citronellolis* (GmR) and treated with antibiotic cocktails (ciprofloxacin 100 μg/ml + carbenicillin 500 μg/ml). After 24 h, fluorescent gut‐associated colonies were checked; disruption and plating of intestinal contents indicated if all live bacteria were cleared out from the worm intestine.

### Larval yield assay

In these experiments, we chose to measure fecundity in individual worms rather than in groups of hermaphrodites on shared plates, to avoid statistical issues associated with taking average measurements on long‐tailed distributions and thereby to increase our power to detect differences between groups. N2 wild‐type worms were synchronized according to standard protocols (Stiernagle, 2006), and L1 worms were transferred to 10 cm NGM plates containing OP50 only, OP50 + *P. cellulosa* (50 μl 10× bacterial suspension from 48 h culture in 10 ml ATCC 2720, grown at room temperature with shaking), OP50 + *B. subtilis* (prepared identically to *P. cellulosa* using LB as growth medium). Worms were incubated on plates at 25°C for 46 h, then washed off plates using M9 worm buffer +0.1% Triton X‐100, rinsed 3× to remove the bulk of extracellular bacteria, and picked as single worms into individual wells of a 384‐well plate containing 25 μl S medium +0.25× heat‐killed OP50 (from 50× stock, cells concentrated 50× from stationary phase) + 25 μg/ml gentamycin (to prevent external growth of bacteria) ± carbon (glucose or carboxymethylcellulose). For each combination of bacteria and carbon source, 24 worms were assayed in each experiment. After 48 h, larvae were counted in individual wells using 4× magnification and white light illumination on a Ti–E inverted microscope. This time point was chosen because at 24 h, there were still a large number of zero larval counts across conditions, as many worms had not yet started laying eggs and early eggs were still in the process of hatching, and dehydration in the plate began to become an issue by 72 h.

### Assay for nutritional status using an integrated fluorescent reporter

We used transgenic worms expressing the *fat‐7p*::GFP reporter fluorescent reporter as an indicator of host nutritional status (Lee *et al*, 2015). *fat‐7p*::GFP worms were synchronized according to standard protocols and grown to day 0 adults on *pos‐1* plates to prevent reproduction, then transferred to 6 cm NGM plates containing live *E. coli* OP50 *P. cellulosa*, *P. cellulosa* was grown at 25°C, in 5 ml ATCC 2720 then pelleted at 9,056 *g* for 2 min and resuspended in S medium to concentrate the bacteria prior to seeding NGM plates with pre‐existing OP50 lawns (50 μl of each suspension per plate). Worms were allowed to feed on these plates for 24 h at 25°C to allow bacterial colonization.

Nutritional status was read on a BioSorter large object sorter using the 250 micron nozzle. Adult worms were transferred from plates to individual wells of a 24‐well plate in S medium with heat‐killed *E. coli* (to provide nitrogen and other nutrients) + gentamycin 10 μg/ml (to prevent growth of bacteria outside the worm) ± glucose or CMC at the indicated concentrations, covered with a BreatheEasy gas‐permeable membrane, and incubated for 24 h at 25°C with shaking at 200 rpm. After incubation, worms were washed to remove heat‐killed bacteria, and total GFP fluorescence per worm was measured on the BioSorter. Worms were gated based on extinction and time of flight (TOF) to isolate full‐sized adult worms in these data, due to imperfect penetrance of the *pos‐1* reproductively sterile phenotype in some experiments.

### High‐throughput carbon source utilization screen

Eighteen species were inoculated from frozen glycerol stock into 2 ml of their preferred rich medium (see Appendix Table S1), and grown in a shaking incubator at 30°C and 300 rpm for 48 h. Fast‐growing species exceeding OD ~1.0 in the first 24 h of this period were diluted 1:1,000 into fresh rich media to grow for the remaining 24 h. All cultures were then centrifuged at 1,370 *g* for 5 min, then resuspended in an M9 media formulation lacking any carbon source. OD600 was measured in a 96‐well plate on a SpectraMax M5 platereader, and culture densities were normalized to OD 1.0. These cultures were diluted 1:20 into deep 96‐well plates containing 400 μl of 12 different M9 media formulations, each with a different carbon source at 0.5% concentration (arabinose, cellobiose, carboxymethyl cellulose, ethanol, glucose, lactose, raffinose, soluble starch, sucrose, xylan hemicellulose, and xylose, as well as a no‐carbon control). Cultures were grown at 30°C and 900 rpm for 48 h. OD600 was measured again, and the M9 no‐carbon control cultures were used to subtract out apparent growth that may have occurred from residual rich media or absorbance changes independent of carbon utilization. Triplicate assays were performed on different days, with pairwise correlations of *r* ^2^ ≥ 0.87 between days.

### Antibiotic susceptibility screen

Four species were grown from frozen glycerol stocks for 48 h in preferred rich media using the same protocol as in the carbon utilization screens. Cultures were centrifuged at 3,500 rpm, and resuspended in fresh rich medium and normalized to an OD600 of 1.0 by diluting with additional rich medium. Normalized cultures were next diluted 1:100 into deep 96‐well plates containing 400 μl rich media dosed with one of seven antibiotics (carbenicillin, chloramphenicol, ciprofloxacin, kanamycin, nalidixic acid, streptomycin, tetracycline, and an ethanol control) at 1×, 5×, and 10× working concentrations. Cultures were grown at 30°C and 900 rpm for 24 h, then OD600 was measured on a SpectraMax M5 plate reader to determine bacteriostatic potential. To determine whether each drug had proved bactericidal, five 5 μl spots of each culture were grown on permissive nutrient agar. Colony counts were binned by order of magnitude (i.e., 0, 1–10, 11–100, lawn of > 100 colonies).

### Engineered cellulolytic *P. putida*

To engineer non‐cellulolytic *P. putida* to cellulolytic bacteria, *P. putida* was transformed with pET24a with ice‐nucleation protein fusion with endoglucanase Cel5A assembled through Gibson assembly. Induction of Cel5A surface display was achieved with 0.1 mM Isopropyl β‐D‐1‐thiogalactopyranoside (IPTG) at OD 1 of bacteria culture. An overnight (16 h) induction at 16°C was conducted before bacteria were collected, and reducing sugar assay from the bacteria was checked. We used *P. putida* because it is a *Pseudomonas* strain, so it serves as a good negative control strain for our cellulolytic bacteria, *P. cellulosa*. Endoglucanase was chosen for these experiments because it hydrolyzes cellulose by cutting randomly on the cellulose chain. Ice‐nucleation protein is a bacterial surface display tag that has been used to demonstrate the translocation of enzymes across the *P. putida* surface (Yang *et al*, 2007). *P. putida* was transformed with the pET24a‐INP‐Endoglucanase plasmid, which had been sequenced prior to transformation, and enzyme activity was checked after protein expression. Since endoglucanase only works by being exposed to the substrate cellulose, the activity of the enzyme should come from the surface‐displayed enzyme.

### Clearance of bacteria from the gut using antibiotics

In order to drive clearance of bacteria from the gut, we screened antibiotics to develop a cocktail with efficacy against all species in our consortium. *In vitro* tests identified a combination of 50 μg/ml carbenicillin and 10 μg/ml ciprofloxacin that should have proven bactericidal to a broad range of species (Appendix Fig S5). However, during *in‐vivo* experiments, we found that 10× higher concentrations of antibiotics were needed to effectively kill bacteria residing in the gut (Appendix Fig S5).

## Author contributions

**Qing Sun:** Data curation; formal analysis; validation; investigation; visualization; methodology; writing – original draft; project administration. **Nic M Vega:** Data curation; formal analysis; validation; investigation; visualization; methodology; writing – original draft; project administration. **Bernardo Cervantes:** Data curation; formal analysis; methodology. **Christopher P Mancuso:** Data curation; formal analysis; visualization; methodology; writing – original draft. **Ning Mao:** Data curation; methodology. **Megan N Taylor:** Data curation; methodology. **James J Collins:** Conceptualization; resources; supervision; funding acquisition; methodology; project administration; writing – review and editing. **Ahmad S Khalil:** Conceptualization; resources; supervision; funding acquisition; methodology; project administration; writing – review and editing. **Jeff Gore:** Conceptualization; resources; supervision; funding acquisition; methodology; project administration; writing – review and editing. **Timothy K Lu:** Conceptualization; resources; supervision; funding acquisition; methodology; project administration; writing – review and editing.

## Disclosure and competing interests statement

TKL is a co‐founder of Senti Biosciences, Synlogic, Engine Biosciences, Tango Therapeutics, Corvium, BiomX, Eligo Biosciences, Bota.Bio, and NE47Bio. TKL also holds financial interests in nest.bio, Armata, IndieBio, MedicusTek, Quark Biosciences, Personal Genomics, Thryve, Lexent Bio, MitoLab, Vulcan, Serotiny, Pulmobiotics, Provectus Algae, Invaio, and NSG Biolabs. JJC is a co‐founder of Senti Biosciences and Synlogic. ASK is a scientific advisor for and holds equity in Senti Biosciences and Chroma Medicine, and is a co‐founder of Fynch Biosciences and K2 Biotechnologies. BC is a co‐founder of Concerto Biosciences.

## Supporting information



Appendix

## Data Availability

Data supporting the findings of this study are available in the Appendix.
