# Supplementary material for: Enhancing nutritional niche and host defenses by modifying the gut microbiome
Source: Mol Syst Biol. 2022 Nov 15;18(11):e9933. doi: 10.15252/msb.20209933 (PMC9664710; doi:10.15252/msb.20209933)
Supplement: Supplementary file 1 — Appendix [file MSB-18-e9933-s001.pdf]

Enhancing nutritional niche and host defenses by modifying the gut microbiome

TABLE OF CONTENTS

	Page
Appendix Figure S1.	2
Appendix Figure S2.	3
Appendix Figure S3.	4
Appendix Figure S4.	5
Appendix Figure S5.	6
Appendix Figure S6.	7
Appendix Figure S7.	8
Appendix Figure S8.	9
Appendix Table .S1.	10
Appendix Table.S2.	11
Additional References	13

Num.	Genus	Species / Strain	Arabinose	Cellobiose	Carboxymethyl Cellulose	Ethanol	Glucose	Lactose	Raffinose	Starch	Sucrose	Xylan	Xylose
**BRICS 09**	*Bacillus*	*subtilis*	0.04	0.06	0.01	0.00	0.07	0.01	0.02	0.09	0.07	0.10	0.04
**BRICS 23**	*Lactobacillus*	*plantarum*	0.01	0.06	0.00	0.01	0.06	0.01	0.01	0.05	0.05	0.03	0.01
**BRICS 26**	*Pseudomonas*	*cellulosa*	1.54	1.46	0.07	0.01	1.47	1.37	0.01	1.37	0.13	1.40	1.47
**BRICS 27**	*Pseudomonas*	*putida* (cel5a)	0.01	0.02	0.04	0.93	1.25	0.02	0.01	0.07	0.24	0.02	0.04
**BRICS 35**	*Achromobacter*	myb9	0.01	0.02	0.00	0.06	0.03	0.02	0.02	0.04	0.01	0.04	0.02
**BRICS 36**	*Acinetobacter*	myb10	0.02	0.02	0.06	1.20	0.04	0.02	0.03	0.04	0.01	0.02	0.01
**BRICS 37**	*Pseudomonas*	myb11	1.00	0.02	-0.01	0.02	1.02	0.01	0.02	0.11	0.28	0.07	0.40
**BRICS 38**	*Ochrobactrum*	myb14	0.24	0.04	0.02	0.14	0.31	0.01	0.02	0.23	0.25	0.10	0.24
**BRICS 39**	*Arthrobacter*	myb27	1.35	1.36	0.01	0.70	1.27	1.37	0.92	0.93	1.19	1.27	0.48
**BRICS 41**	*Rhodococcus*	myb53	0.01	0.01	0.12	0.93	0.32	0.04	0.01	0.15	1.18	0.06	0.00
**BRICS 42**	*Bacillus*	myb56	0.00	0.14	0.01	0.01	0.07	0.00	0.02	0.12	0.11	0.10	0.01
**BRICS 43**	*Stenotrophomonas*	myb57	0.00	0.00	0.01	0.01	0.01	0.02	0.01	0.02	0.00	0.00	0.01
**BRICS 44**	*Ochrobactrum*	myb237	0.25	0.01	0.01	0.00	0.11	0.01	0.01	0.11	0.15	0.08	0.17
**BRICS 45**	*Stenotrophomonas*	myb238	0.02	1.03	0.00	0.01	1.01	0.00	0.00	0.70	0.22	0.17	0.03
**BRICS 46**	*Ochrobactrum*	myb71	0.49	0.00	0.01	-0.02	0.33	0.01	0.03	0.23	0.27	0.12	0.38
**BRICS 48**	*Chryseobacterium*	myb120	0.00	0.02	0.00	0.00	0.02	0.00	0.00	0.02	0.02	0.02	0.01
**BRICS 49**	*Comamonas*	myb131	0.01	0.01	0.00	0.01	0.01	0.01	0.01	0.02	0.01	0.01	0.01
**BRICS 50**	*Sphingobacterium*	myb181	0.22	0.46	0.00	0.00	0.46	0.43	0.30	0.50	0.37	0.46	0.06

Appendix Figure S1. High-throughput *in vitro* carbon source growth screen. Twenty-nine species were tested (including *C. elegans* gut microbes) with 11 carbon sources in M9 minimal media. ΔOD600 vs. no-carbon control over 48 h agrees between 3 replicates: ($R^2 \geq 0.87$).

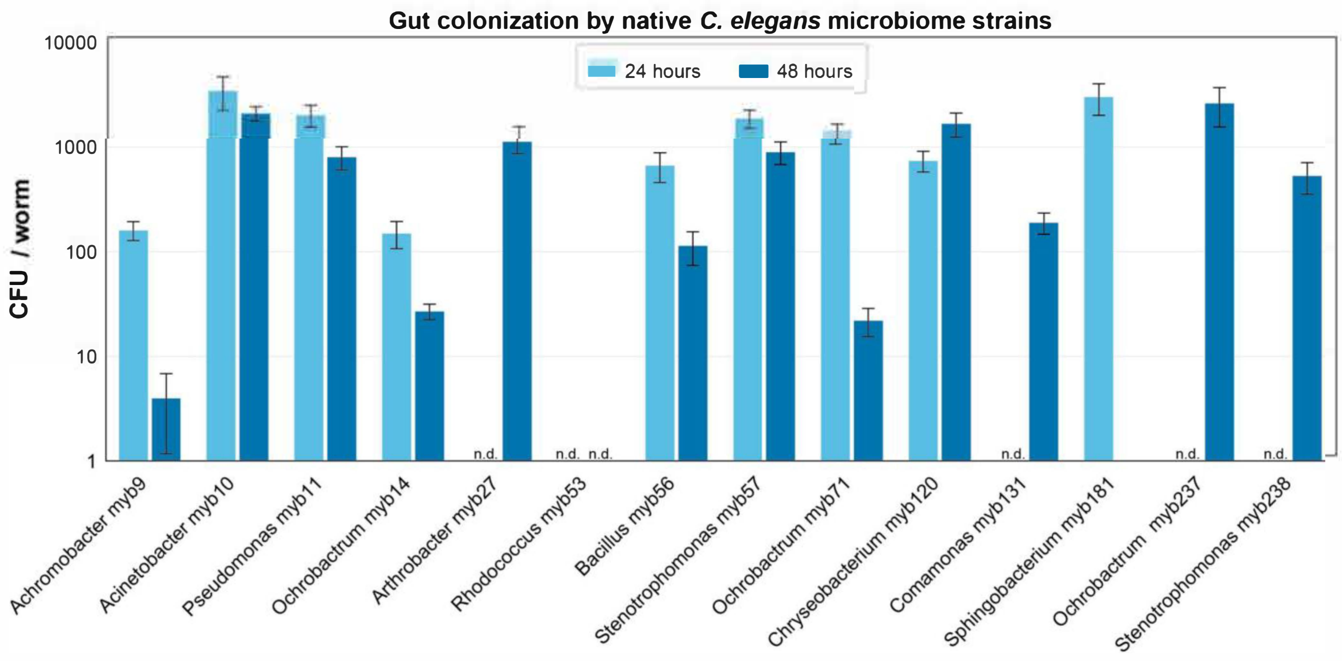

N

Appendix Figure S2. *C. elegans* native microbes colonize the worm gut. Sixteen native microbes (with strain names in Appendix Table S1) were each grown independently in LB media at 25℃ for 48 h, diluted 1:10 from stationary phase and used to colonize sterile N2 adult worms by feeding in liquid S medium at this concentration for 24 h. After the initial feeding period, worms were removed from culture; a batch digest of 50 worms was performed (Time 0) to determine average CFU/worm for each microbial species, and the remaining worms were washed thoroughly and transferred to S medium + 1X heat-killed OP50 and incubated for a further 48 h to determine persistence, after which a second batch digest (50 worms) was performed. Error bars represent mean ± SD of count error.

Num.	9b	23a	26a	30a	35a	35b	36a	37a	37b	37c	38a	38b	39a	41a	42a	43a	44a	44b	45a	46c	48a	49a	50a
**BRICS 09**	23	0	0	0	0	0	0	5.7	0	0	0	0	0	0	0	0	0	0	0	0	0	0	0
**BRICS 23**	7.5	15	0	0	0	0	0	5	0	0	0	0	0	0	0	0	0	0	0	0	0	0	0
**BRICS 26**	5	0	25	0	0	5	0	12	5	0	0	0	0	0	0	5	0	9.1	0	0	0	0	5
**BRICS 27**	5	0	10	0	0	0	5	26	14	5	8.5	0	5	0	5	8.2	5	5	5	7.7	0	0	5
**BRICS 29**	0	0	5	0	0	0	0	26	12	5	5	0	0	5	0	7.8	5	5	5	5.5	0	0	0
**BRICS 30**	0	0	0	7.4	0	0	0	5	5	0	0	0	0	5	0	0	0	0	0	0	0	0	0
**BRICS 33**	0	0	0	0	0	0	0	0	0	0	5	0	0	8.1	0	0	0	0	0	0	0	0	0
**BRICS 35**	0	5	7.7	0	14	23	0	5	16	0	11	0	0	5	0	7	5	9.5	5	8.9	0	0	5
**BRICS 36**	0	0	0	0	0	0	24	0	0	0	0	0	0	0	0	0	0	5	0	0	0	0	0
**BRICS 37**	0	0	0	0	5	5	5	25	9.6	26	9.3	0	5	0	5	5	5	13	5	7.8	0	0	5
**BRICS 38**	0	0	0	0	5	0	0	8.1	5	5	21	5	0	0	0	0	20	6.3	0	0	0	0	0
**BRICS 39**	0	0	0	0	5	0	0	0	5	0	14	0	14	7	0	0	0	5	7.8	5	0	0	0
**BRICS 41**	0	0	0	0	0	0	0	0	5	0	7.4	0	6.6	19	0	0	0	0	0	0	0	0	0
**BRICS 42**	0	0	0	0	0	0	0	0	0	0	0	0	0	0	22	0	0	0	0	0	0	0	0
**BRICS 43**	0	0	0	0	5	5	5	5	8.2	0	12	0	5	0	13	6.7	5	6.1	5	5	0	0	0
**BRICS 44**	0	0	0	0	0	0	0	5	0	5	12	0	0	0	5	0	5	22	0	0	0	0	0
**BRICS 45**	0	0	0	0	0	0	0	5	6.1	0	8.5	5	5	0	5	19	0	5	8.1	5	0	0	0
**BRICS 46**	0	0	0	0	5	0	0	5	5	5.7	22	0	0	0	5	0	0	5	0	5	0	0	0
**BRICS 48**	0	0	0	0	0	0	0	5	0	0	0	0	0	0	0	0	5	0	0	0	0	0	0
**BRICS 49**	0	0	5	0	5	0	5	8	10	5	11	0	5	6.7	0	7.8	0	5	6.5	6.7	0	0	5
**BRICS 50**	0	0	0	0	0	0	5	0	0	0	0	0	0	5	0	0	0	0	0	0	0	0	0

Appendix Figure S3. High-throughput screen of orthogonal qPCR identification primers for accurate identification of colonized bacteria. Twenty-nine species (including *C. elegans* gut microbes) were tested with 48 primers, with 1 + primer for each unique species, targeted to an am1otated gyrB gene from closely related species. Displayed values indicate ΔCt compared to a no-template control signal that arose at approximately 35 cycles.

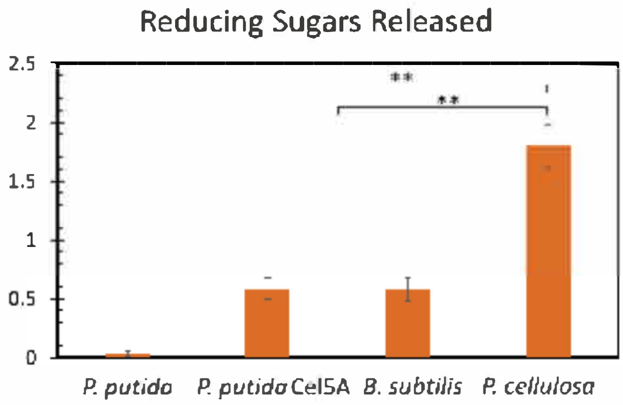

Appendix Figure S4. Quantification of cellulose-hydrolyzing capabilities of natural (*P. putida, B. subtilis, P. cellulosa)* and engineered (*P. putida* Cel5A) soil organisms. $^{**}P < 0.01$, $^{***}P < 0.001$, Student's t test. Error bars represent 95% confidence intervals of the mean.

Num.	Genus	Species	Ethanol (control)			Carbenicillin			Chloramphenicol			Ciprofloxacin		
			0.1%	0.5%	1.0%	50	250	500	34	170	340	10	50	100
**BRICS 09**	*Bacillus*	*subtilis*	1.22	1.27	1.20	0.00	0.00	0.00	0.01	0.04	0.03	0.01	0.04	0.04
**BRICS 23**	*Lactobacillus*	*plantarum*	1.64	1.61	1.58	0.08	0.09	0.08	0.07	0.07	0.07	1.22	1.34	1.18
**BRICS 26**	*Pseudomonas*	*cellulosa*	0.23	0.07	0.06	0.18	0.22	0.08	0.11	0.23	0.08	0.02	0.03	0.03
**BRICS 27**	*Pseudomonas*	*putida* (cel5a)	0.88	1.22	1.77	0.71	0.69	0.40	0.79	0.26	0.04	0.02	0.05	0.05

Num.	Kanamycin			Nalidixic Acid			Streptomycin			Tetracycline			
	50	250	500	30	150	300	50	250	500	10	50	100	Conc. (ug/mL)
**BRICS 09**	0.01	0.04	0.04	0.02	0.05	0.04	0.01	0.05	0.04	0.01	0.04	0.03	
**BRICS 23**	1.52	1.57	1.27	1.61	1.59	1.48	0.21	1.55	1.33	0.11	0.12	0.11	
**BRICS 26**	0.02	0.08	0.06	0.02	0.04	0.03	0.03	0.10	0.09	0.07	0.09	0.09	
**BRICS 27**	0.61	0.05	0.04	0.72	0.08	0.06	0.02	0.06	0.05	0.01	0.07	0.05	

Num.	Genus	Species	Ethanol (control)			Carbenicillin			Chloramphenicol			Ciprofloxacin		
			0.1%	0.5%	1.0%	50	250	500	34	170	340	10	50	100
**BRICS 09**	Bacillus	subtilis	3	3	3	2	0	0	3	0	0	1	0	0
**BRICS 23**	Lactobacillus	plantarum	2	2	2	2	0	0	2	2	1	2	2	2
**BRICS 26**	Pseudomonas	cellulosa	1	1	0	1	1	0	0	0	0	0	0	0
**BRICS 27**	Pseudomonas	putida (cel5a)	3	3	3	3	3	2	3	3	1	0	0	0

Num.	Kanamycin			Nalidixic Acid			Streptomycin			Tetracycline			
	50	250	500	30	150	300	50	250	500	10	50	100	Conc. (ug/mL)
**BRICS 09**	0	0	0	2	0	0	2	0	0	2	0	0	
**BRICS 23**	2	2	2	2	2	2	2	2	2	2	1	1	
**BRICS 26**	0	0	0	0	0	0	1	0	0	1			
**BRICS 27**	3	0	0	3	0	0	0	0	0	1	2	2	

3	Lawn or >1000 colonies
2	Faint lawn or >100 colonies
1	<100 colonies
	no colonies

Appendix Figure S5. Large-scale *in vitro* screen to identify antibiotic cocktail for bacteriostatic and bactericidal treatments. (A) Optical density and (B) colony counts were both checked to determine bacteriostatic and bactericidal potential, respectively.

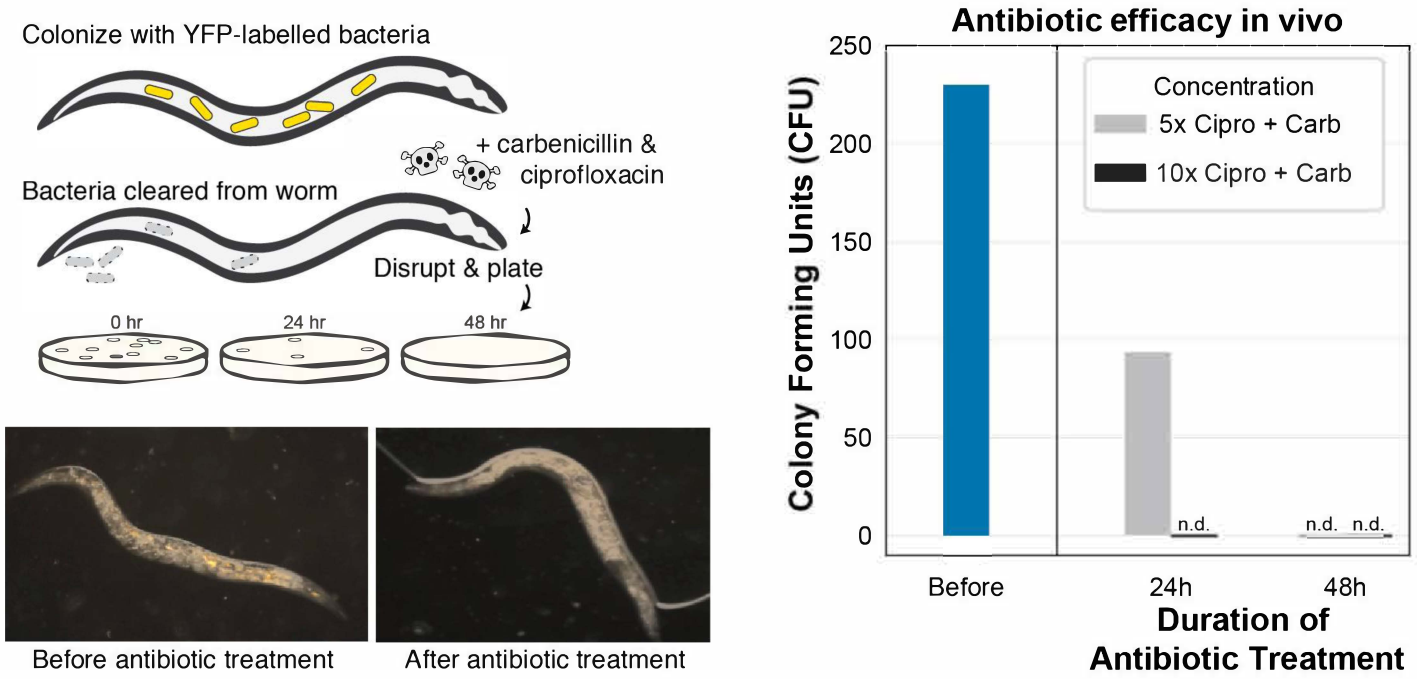

Appendix Figure S6. Microscope images and plate assays to validate removal of bacteria colonized in worm gut. (Top left) Assay design. (Right) For plate assays, *C. elegans* were disrupted and plated on LB media plates for overnight culturing to check on colonized bacteria. (Bottom left) For microscope images, *C. elegans* with yellow fluorescence protein-expressing *P. citronellolis* before and after antibiotic treatment were checked under a fluorescence microscope for fluorescent bacteria in the gut.

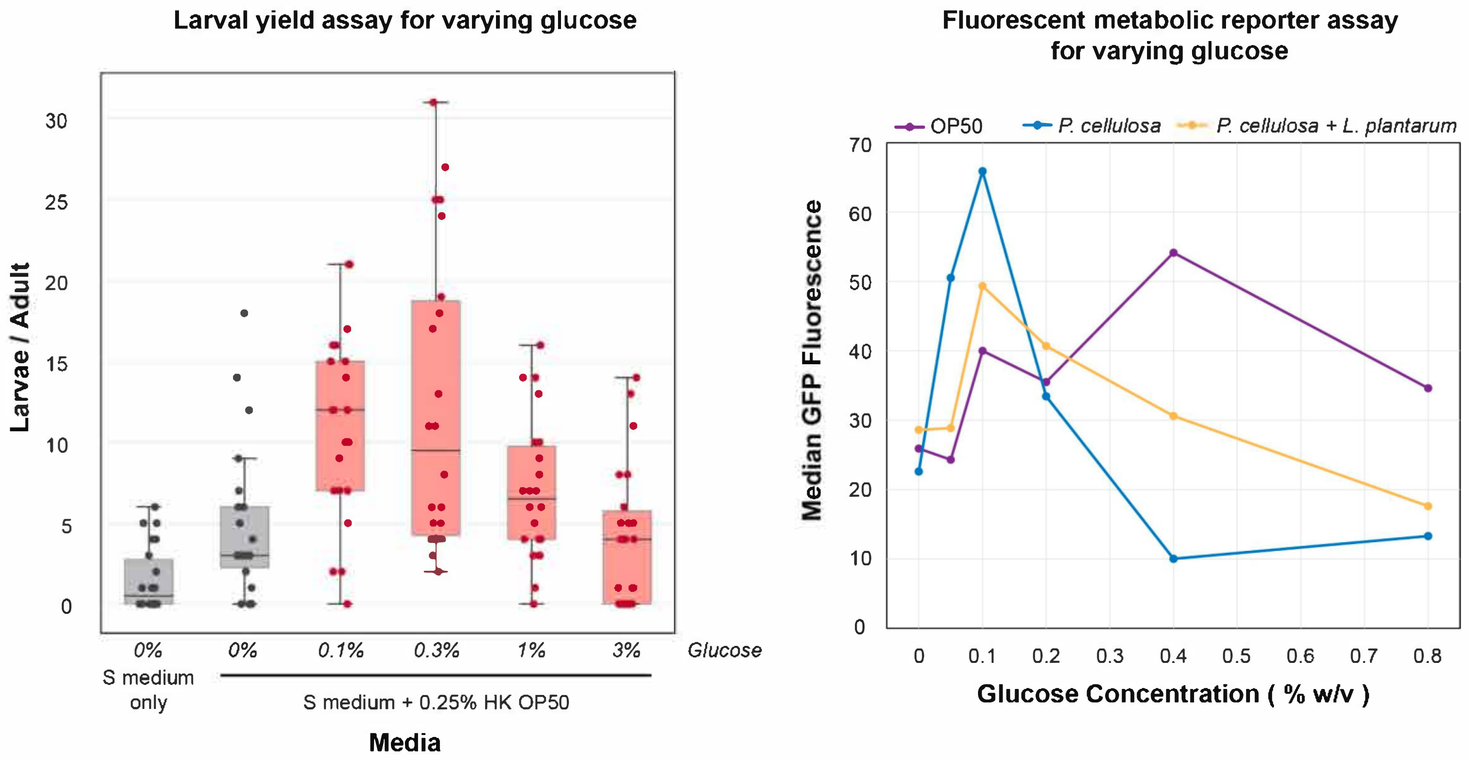

Appendix Figure S7. Optimal carbon source concentrations for nutritional benefit experiments. (Left) In larval yield experiments, an optimum concentration of ~0.1%-0.3% glucose was determined. (Right) Results from the *fat-7p:* :GFP fluorescent reporter of host nutritional status (median GFP fluorescence per individual adult worm, ~1000 worms/condition) are consistent with the results of larval yield assays. It is plausible that the loss of benefit to the host at higher glucose concentrations is due to negative effects from osmolarity of the solution at these high concentrations.

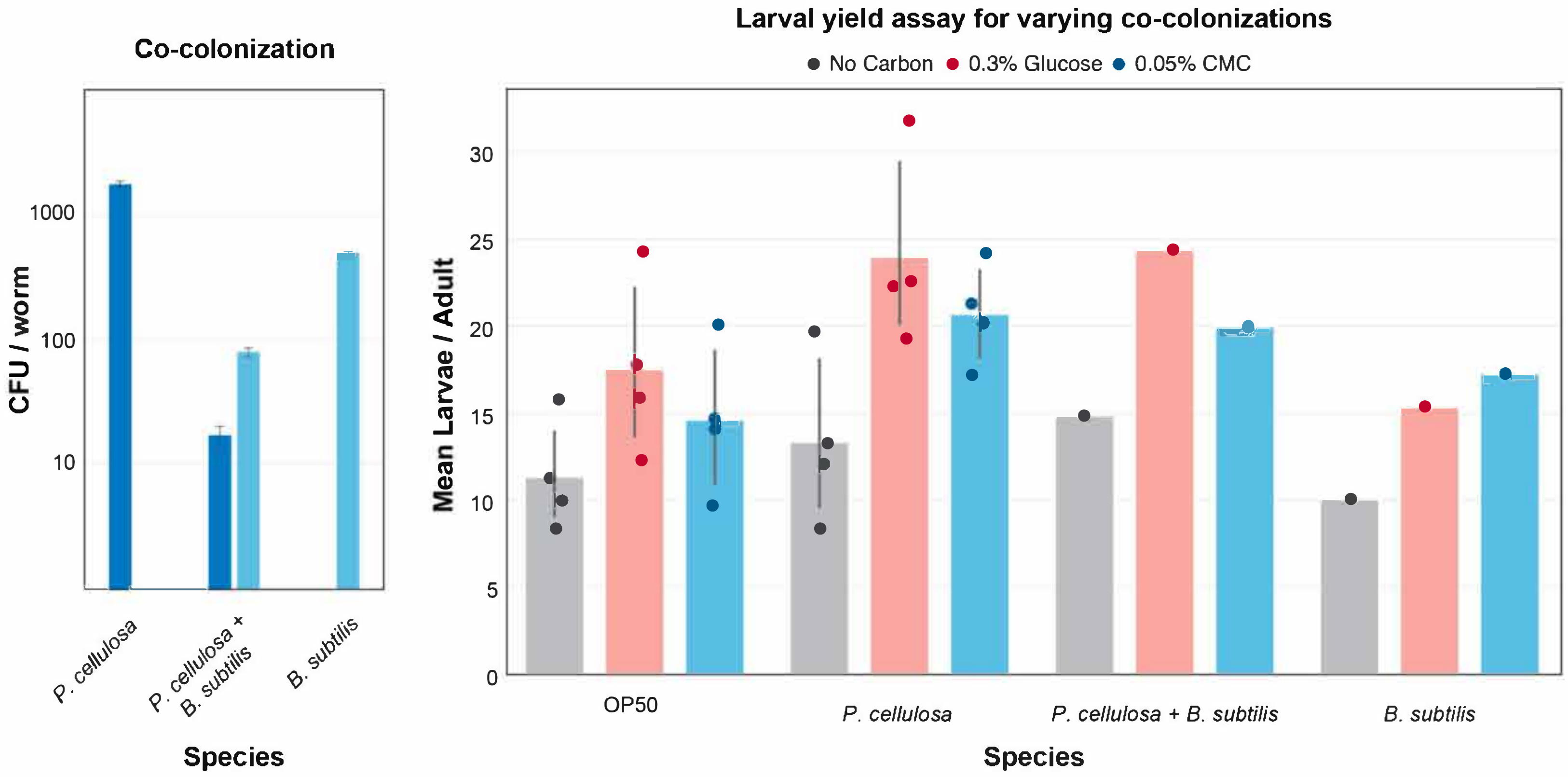

Appendix Figure S8. Co-colonization of the host with *Pseudomonas cellulosa* and *Bacillus subtilis* provides no additional nutritional benefit. (Left) Co-colonization leads to reduced overall densities of bacteria in the gut, and particularly to suppression of *P. cellulosa,* indicating that this efficient cellulose degrader is out-competed by *B. subtilis.* (Right) Consistent with these results, co-colonization does not increase the benefit of CMC supplementation to the host (wild-type N2 worms), as measured by larval output.

Appendix Table S1. Bacterial strains used in this study.

Number	Genus	Species/Subspecies	Strain Designation	Source	Growth Media
**BRICS 01**	*Enterobacter*	*aerogenes*	13048	ATCC	NB
**BRICS 02**	*Pseudomonas*	*aurantiaca*	33663	ATCC	NB
**BRICS 03**	*Pseudomonas*	*chlororaphis*	9446	ATCC	NB
**BRICS 04**	*Pseudomonas*	*citronellolis*	13674	ATCC	NB
**BRICS 05**	*Pseudomonas*	*fluorescens*	13525	ATCC	NB
**BRICS 06**	*Pseudomonas*	*putida*	12633	ATCC	NB
**BRICS 07**	*Pseudomonas*	*veronii*	700474	ATCC	NB
**BRICS 08**	*Serratia*	*marcescens*	13880	ATCC	NB
**BRICS 09**	*Bacillus*	*subtilis 168*	23857	ATCC	NB
**BRICS 23**	*Lactobacillus*	*plantarum*	14917	ATCC	MRS
**BRICS 26**	*Pseudomonas*	*cellulosa*	55703	ATCC	ATCC2720
**BRICS 27**	*Pseudomonas*	*putida*	KT2440 (cel5a)	This Study	LB + Kanamycin
**BRICS 29**	*Pseudomonas*	*putida*	KT2440 (bglS)	This Study	LB + Kanamycin
**BRICS 30**	*Lactococcus*	*lactis subsp. Cremoris*	MG1363	ATCC	GM17
**BRICS 33**	*Bacillus*	*subtilis subsp. Spizizenii*	6633	ATCC	NB
**BRICS 35**	*Achromobacter*	*sp. F32*	myb9	Dirksen et al 2016	NB
**BRICS 36**	*Acinetobacter*	*sp. LB BR 12338*	myb10	Dirksen et al 2016	NB
**BRICS 37**	*Pseudomonas*	*lurida*	myb11	Dirksen et al 2016	NB
**BRICS 38**	*Ochrobactrum*	*sp. BS30*	myb14	Dirksen et al 2016	NB
**BRICS 39**	*Arthrobacter*	*aurescens*	myb27	Dirksen et al 2016	NB
**BRICS 41**	*Rhodococcus*	*erythropolis PR4*	myb53	Dirksen et al 2016	NB
**BRICS 42**	*Bacillus*	*sp. SG20*	myb56	Dirksen et al 2016	NB
**BRICS 43**	*Stenotrophomonas*	*sp. R-41388*	myb57	Dirksen et al 2016	NB
**BRICS 44**	*Ochrobactrum*	*pseudogrignonense*	myb237	Dirksen et al 2016	NB
**BRICS 45**	*Stenotrophomonas*	*acidaminiphila*	myb238	Dirksen et al 2016	NB
**BRICS 46**	*Ochrobactrum*	*sp. R-26465*	myb71	Dirksen et al 2016	NB
**BRICS 48**	*Chryseobacterium*	*sp. CHNTR56*	myb120	Dirksen et al 2016	NB
**BRICS 49**	*Comamonas*	*sp. 12022*	myb131	Dirksen et al 2016	NB
**BRICS 50**	*Sphingobacterium*	*faecium*	myb181	Dirksen et al 2016	NB
**NA**	*Escherichia*	*coli*	OP50	Caenorhabditis Genetic Center	NB

Appendix Table S2. qPCR Primers tested in this study.

Primer Num.	Sequence	Target Species	Pair
pRCM224	GTCACCCGCTGGGTTAATCA	1	a
pRCM225	CTGCAGCTCGCTGTTTTCAC	1	a
pRCM226	TCTGGGAACAGACCTACGTTCA	2	a
pRCM227	GATGTTCTTGAAGGTCTCGCTGGA	2	a
pRCM228	GGACAGTTCACGAATCCGCTT	2	b
pRCM229	ACCCAGATTCACTTCAAGGCTTCTA	2	b
pRCM230	ACGCAACCGTAAGACCCAG	3	a
pRCM231	CCGACCTCTTGCGAGGAAAT	3	a
pRCM232	TCTGGGAGCAGATCTATCGTC	4	a
pRCM233	CCAGCTGAAGTGGATATTGGTAAA	4	a
pRCM249	CTACAAGGTTTCCGGTGGCT	4	b
pRCM250	GATCTGCTCCCAGACCTTGC	4	b
pRCM251	ACAAGCTGGTCTCCTCCGA	4	c
pRCM252	CTTGGCTTCGTTGGGGTTCT	4	c
pRCM255	CGAAGGCAAGGTCTGGGAG	4	d
pRCM256	GATGCTGGTGAAGGTCTCGT	4	d
pRCM257	GAAGGCAAGGTCTGGGAGC	4	e
pRCM258	TGCTGGTGAAGGTCTCGTTG	4	e
pRCM234	ATGAAAATCGTTGGCGACAG	5	a
pRCM235	CCGGAGTTGAGGAAGGACAG	5	a
pRCM243	GAATCCACGGGTACGCAGAT	5	b
pRCM244	CACCGGAGTTGAGGAAGGAC	5	b
pRCM245	ATGGCGGTACTCACTTGGTG	5	c
pRCM246	GGCGACTTTGTGCTTCTTGG	5	c
pRCM247	ACTCACTTGGTGGGTTTCCG	5	d
pRCM248	ATAATCGCGGTCAGGCCTTC	5	d
pRCM253	GAATCCACGGGTACGCAGAT	5	e
pRCM254	CACCGGAGTTGAGGAAGGAC	5	e
pRCM259	AAAACCCTCAAGCGTCTTTCG	5	f
pRCM260	AACGCCTTCAGCCAAATGTC	5	f
pRCM261	GATAAGGCGCAGATGGACATT	5	g
pRCM262	AACCATGGGAGGTCGTTTCA	5	g
pRCM236	AAGTGGAAATCACCTCCCACG	6	a
pRCM237	GTACGTAAGCACCTTCACCCAA	6	a
pRCM238	TAAACGCCCTCTCCGAATTGC	7	a
pRCM239	GCTTTCGCCAACAACCTTCATC	7	a
pRCM240	GTGCACGAACAAACTTACAGCC	8	a
pRCM241	GGTCACATTGGTAAAGGTCTGGT	8	a
pRCM307	CGCCAAACCTATAAACGCGG	9	b
pRCM308	TCAGGGTCCGGGACAAAATG	9	b

pRCM349	GTGGGACGCATGAAGAAGGT	23	a
pRCM350	TCTTCGCCAGATAGGTTCGC	23	a
pRCM311	AGACAAGCTGGTGTCTTCCG	26	a
pRCM312	CCACTGCCTTGGCATCATTG	26	a
pRCM351	GCAGACGAATATGACGCCAG	30	a
pRCM352	GGTGCAACCCTTCTTTTGAGG	30	a
prCM407	AGTTCAGCAGCCAGACCAAG	35	a
prCM408	TTCTCGAGCAGCCAGGATTC	35	a
prCM415	GAACAACGGCGTCAAGATCC	35	b
prCM416	TTGGCGCGGTTGATGTATTC	35	b
prCM440	GCGATTGAAACAACGCAACC	36	a
prCM441	CGCGGGAAATAAATCGTCGT	36	a
prCM399	CCGAAGGTCTGGCCAAGAAG	37	a
prCM400	TCCGGTACCTTCACCGAGAT	37	a
prCM403	ATCGTTACGACCGCAACCTG	37	b
prCM404	AGTGCATCCAGCCCTTCG	37	b
prCM401	AAGGTCTGGCCAAGAAGCAT	37	c
prCM402	GATCCGGTACCTTCACCGAG	37	c
prCM397	AGACCATATGATGCAGGCCG	38	a
prCM398	CGCGGATTCCATTCGTGTTC	38	a
prCM442	CTTTGCGACGAAGTACAGGC	38	b
prCM443	TATGGCACTGCTCGGTTCTG	38	b
prCM448	GCTTTCGAAACCGAGGACAC	39	a
prCM449	GTGTTTGCGTACGTGTGGAC	39	a
prCM450	GCTCCAACGATCCGGAGAAG	41	a
prCM451	CTGGTACATCGAGTCTCGGC	41	a
prCM446	ACACCATGCAAACCACCAGA	42	a
prCM447	ATGGGACGTCCTGCTGTAGA	42	a
prCM454	TGCTGGTGGATGTGTTCCAG	43	a
prCM455	GTTTGGTGGTGGTTTCCAGC	43	a
prCM464	AGGACAAGCTCGTTTCCTCG	44	a
prCM465	TTCGACGATGGTCTTGCCAC	44	a
prCM466	TCAAGCTTACAGACCGTCGC	44	b
prCM467	AGCGACTTCTTGCTCTGGTC	44	b
prCM468	GCTGATGGAGGAAGCCAAGA	45	a
prCM469	GTATCGGGGTTGACCGTGG	45	a
prCM452	AAGAAGCCACGCTGGAAATG	46	c
prCM453	CGTGTGAAGACCCGCCAG	46	c
prCM458	CCGACGAAAGAGAAAGGTTGG	48	a
prCM459	TGGATTCCCTGCTTCCATCG	48	a
prCM460	GTCAGCTCCGAAGTCCGTG	49	a
prCM461	TCGACAATCTTGTTGCAGAGAATC	49	a
prCM462	TGGTGTAACCCTTCGCCAC	50	a
prCM463	CGTGCGCAAGAATATGACGC	50	a

Additional References

Lee, D., Jeong, D.E., Son, H.G., Yamaoka, Y., Kim, H., Seo, K., Khan, A.A., Roh, T.Y., Moon, D.W., Lee, Y., et al. (2015). SREBP and MDT-15 protect C. Elegans from glucose-induced accelerated aging by preventing accumulation of saturated fat. Genes Dev. *29*, 2490–2503.

Rual, J.F., Ceron, J., Koreth, J., Hao, T., Nicot, A.S., Hirozane-Kishikawa, T., Vandenhaute, J., Orkin, S.H., Hill, D.E., van den Heuvel, S., et al. (2004). Toward improving Caenorhabditis elegans phenome mapping with an ORFeome-based RNAi library. Genome Res. *14*, 2162–2168.

Stiernagle, T. (2006). Maintenance of C. elegans. WormBook.

Sun, Q., and Chen, W. (2016). HaloTag mediated artificial cellulosome assembly on a rolling circle amplification DNA template for efficient cellulose hydrolysis. Chem. Commun. *52*, 6701–6704.

Yang, C., Cai, N., Dong, M., Jiang, H., Li, J., Qiao, C., Mulchandani, A., and Chen, W. (2008). Surface Display of MPH on Pseudomonas putida JS444 Using Ice Nucleation Protein and Its Application in Detoxification of Organophosphate, Biotechnol. Bioeng. *99*, 30-37.
