# Supplementary material for: Enhancing nutritional niche and host defenses by modifying the gut microbiome
Source: Mol Syst Biol. 2022 Nov 15;18(11):e9933. doi: 10.15252/msb.20209933 (PMC9664710; doi:10.15252/msb.20209933)
Supplement: Supplementary file 2 [file MSB-18-e9933-s002.pdf]

molecular systems biology

Enhancing nutritional niche and host defenses by modifying the gut microbiome

Qing Sun, Nicole Vega, Bernardo Cervantes, Christopher Mancuso, Ning Mao, Megan Taylor, James Collins, Ahmad Khalil, Jeff Gore, and Timothy Lu
DOI: 10.15252/msb.20209933

Corresponding author(s): Timothy Lu (timlu@mit.edu) , Qing Sun (sunqing@tamu.edu)

Review Timeline:

Submission Date:	21st Aug 20
Editorial Decision:	14th Oct 20
Revision Received:	22nd Jun 21
Editorial Decision:	30th Jun 21
Revision Received:	15th Aug 22
Accepted:	14th Oct 22

Editor: Maria Polychronidou

Transaction Report:

(Note: With the exception of the correction of typographical or spelling errors that could be a source of ambiguity, letters and reports are not edited. Depending on transfer agreements, referee reports obtained elsewhere may or may not be included in this compilation. Referee reports are anonymous unless the Referee chooses to sign their reports.)

1st Editorial Decision 14th Oct 2020

Thank you again for submitting your work to Molecular Systems Biology. We have now heard back from the three referees who agreed to evaluate your study. Overall, the reviewers acknowledge that the presented findings seem potentially interesting. However, they raise a series of concerns, which we would ask you to address in a major revision.

As you will see below, reviewer #3 is not supportive and thinks that the study in its current form seems too preliminary in terms of the provided advance. Nevertheless, given the supportive comments of reviewers #1 and #2 we have decided to give you a chance to revise the study and address the issues raised. During our pre-decision cross-commenting process (in which the reviewers are given the chance to make additional comments, including on each other's reports), reviewers #1 and #2 mentioned that the study needs to be more clearly presented from a synthetic biology perspective, to make clear to the readers that there is no attempt to reveal new basic worm biology. The two reviewers also agreed that several of the issues raised by reviewer #3 and referring to the need for including controls, better supporting the conclusions and more carefully interpreting the results need to be addressed.

Without repeating all the issues listed below, some of the more fundamental issues raised by the reviewers are the following:

Reviewer #1:

I enjoyed reading this manuscript. The authors tested a simple yet elegant concept of using a non-native symbiont to alter host diet and response to pathogen invasion. The study is well performed (especially the 14C experiments provide a solid evidence) and well written. I have only two suggestions:

1. Data presentation: Please show the actual data points in the bar plots. Also, the exact p-values should be reported (except when they are below the accuracy limit of the test/algorithm).

2. Figure 1 is nice but its a kind of a "give away" - shouldn't this be more of a summary figure?

Reviewer #2:

This is an interesting manuscript. From an engineering perspective, designing synthetic microbiomes to endow novel functions on the host is exciting. From a scientific perspective, simple models for gut bacterial-host interactions are valuable for unraveling the mechanisms by which gut bacteria interact with hosts, other gut bacteria, invading pathogens, as well as how the host interacts with these organisms.

Overall, I think the work is well done and suitable for publication in MSB. However, I have several concerns about experiments performed, conclusions drawn, and presentation that need to be addressed prior to publication.

Which bacteria in Table S1 are native C. elegans symbionts, and which are soil bacteria? This information should be added to Table S1.

All worm growth/development stages, non-introduced bacterial strains (e.g. OP50), and worm growth media, should be introduced, and the rationale for their use explained, in the main text.

The robust levels of colonization of the CMC digesting bacteria in Figure 2D relative to the native worm symbionts (Figure S2) is impressive, and makes the implications of the study more interesting.

Much more information on the endoglucanase expressing P. Putida is needed. Currently it's just a blurb in the methods. Why was P. Putida chosen as opposed to any other non CMC utilizing bacterium? Why was endoglucanase chosen as opposed to any other carbohydrate active enzyme? What is the ice-nucleation protein and why was it chosen? Were the constructed pET24a plasmids sequence verified prior to or subsequent to transformation (it doesn't sound like it based on the current methods)? Plasmid maps and annotated sequences should be provided.

No evidence is provided to support the claim that Gentamicin treatment kills bacteria outside, but not inside the worm. The conclusion that the cellulose-derived benefit came from gut, but not external bacteria, in Figure 3B relies on this claim. Thus, the evidence for the claim must be added.

The data in Figure 3B do not convincingly support the argument that carbon from microbiome-processed CMC benefit host metabolism. The larval assay used in Figure 3C is far more convincing. I recommend moving Figure 3B to the SI and giving it only a brief discussion in the main text.

Why does addition of CMC result in more inhibition of Salmonella infection when P. cellulosa and L. plantarum are present? Is Pc degrading CMC so that Lp has more glucose to grow, and produce beneficial effects? This result is interesting, but very empirical at this point, and not satisfying given that the value of the approach in the ability to understand what is happening due to the simplicity of the system.

The first 3 sentences of the discussion are more appropriate for the introduction.

I do not understand the arguments the authors are making in the 3rd paragraph of the Discussion. In particular: "The introduction of multiple functionally equivalent taxa (as with P. cellulosa and B. subtilis) does not necessarily increase the functional capacity of the community if there is exploitation competition between strains; this can reduce the functional redundancy that would otherwise be a desirable outcome of this colonization 20 strategy. However, in our experiments, the use of a facultatively interacting taxon (L. plantarum) allowed us to add a new functional capacity (resilience against pathogen invasion) by capitalizing on the particular properties of this facultative species..."

I also don't understand what they are arguing here: "Our results also indicate that nutritional interactions can enhance the performance of the system." Isn't the microbiome-host paradigm a nutritional interaction to a large extent?

The idea of engineering the microbiome to introduce new functionality (final paragraph) is exciting.

Minor
The details of the Congo Red assay should be moved from the Results to the Materials and Methods.

Table S1 did not render well.

Numerous supplemental figures are called out incorrectly in the main text (e.g. Figure S4, Figure S5).

Reviewer #3:

This paper claims to "expand host functionality by modifying the gut microbiome" and to "engineer microbiome communities". Unfortunately, it does not convincingly do either. The paper describes two loosely connected observations that potentially could be the start of two rigorous studies. As presented, there is no major advance or novel insight and the paper is mediocre at best. Conclusions that C. elegans uses cellulose broken down by P. cellulosa are not sufficiently supported by the data. In all experiments, worms are colonized with a mixture of P. cellulosa and E. coli. It is possible that the effects observed are indirect, where either bacteria responds to cellulose breakdown products and this in turn affects the worm. Further, the fact that none of the bacteria that are actually found in the wild in the C. elegans gut can metabolize cellulose makes the whole observation less relevant, at least for C. elegans biology. Finally, the paper is overall poorly constructed, the language and descriptions are often vague, there are numerous incorrect references to figures, and, most critically, it lacks rigor.

Other comments:

The title is a massive overstatement of the presented work.

The cartoons are not necessary.

Controls are frequently missing.

For many experiments it is not clear how many replicates were done.

Glucose as a carbon source further enhanced colonization density because free sugar monomers are an easier to access carbon source (page 4) - this is interpretation not substantiated by data.

There is no evidence that CMC is actually hydrolyzed in the C. elegans gut.

The numbers are very different in figures 2D and 2E: ~700 CFU/worm in 2D, but more than ~3000 in 2E. Also, how are E. coli and P. cellulosa colonies distinguished from each other?

They use Pfat-7::GFP as a "reporter of nutritional status" - what does that mean? The results shown in Figure 3B are not convincing. Also, why not just show images, using the sorter makes no sense for this and is not the standard in the field.

The "reproductive fitness" experiments (normally phrased as "brood size") are not convincing either. On E. coli OP50, worms usually have a brood size of 200 or more. Egg laying dynamics can change depending on nutritional input, so a time course is more useful. Also, there is no reason to do this experiment in 384 well plates, where worms run out of food quickly and larvae are hard to count. Bigger plates (or tubes) with more worms would be more appropriate and is feasible because there are only six conditions tested.

Reviewer #1:

I enjoyed reading this manuscript. The authors tested a simple yet elegant concept of using a non-native symbiont to alter host diet and response to pathogen invasion. The study is well performed (especially the 14C experiments provide a solid evidence) and well written. I have only two suggestions:

We thank the reviewer for their support and constructive comments.

1. Data presentation: Please show the actual data points in the bar plots. Also, the exact p-values should be reported (except when they are below the accuracy limit of the test/algorithm).

We have added the actual data points and p-values in the bar plots, as suggested by the reviewer.

2. Figure 1 is nice but its a kind of a "give away" - shouldn't this be more of a summary figure?

We have moved this figure to the last figure of this manuscript (Fig. 4), so it serves as a summary figure.

Reviewer #2:

This is an interesting manuscript. From an engineering perspective , designing synthetic microbiomes to endow novel functions on the host is exciting. From a scientific perspective, simple models for gut bacterial-host interactions are valuable for unraveling the mechanisms by which gut bacteria interact with hosts, other gut bacteria, invading pathogens, as well as how the host interacts with these organisms.

Overall, I think the work is well done and suitable for publication in MSB. However, I have several concerns about experiments performed, conclusions drawn, and presentation that need to be addressed prior to publication.

We thank the reviewer for their insights and constructive comments.

Which bacteria in Table S1 are native C. elegans symbionts, and which are soil bacteria? This information should be added to Table S1.

We have included the information in Table S1 to label *C. elegans* native bacteria and soil bacteria.

All worm growth/development stages, non-introduced bacterial strains (e.g. OP50), and worm growth media, should be introduced, and the rationale for their use explained, in the main text.

We have made the following changes in the text to explain the experimental design:

- (line 122-129): We used OP50 because *C. elegans* is usually grown monoxenically in the laboratory using *E. coli* strain OP50 as a food source (Brenner et al. 1974). In these experiments, synchronized adult N2 worms were allowed to colonize on solid plates instead of liquid media to allow efficient feeding on these bacteria and therefore increase the rate of colonization. As we intended to use fecundity as one measure of carbon

uptake in these experiments and as *C. elegans* fecundity shows a strong response to starvation (adult reproductive diapause) when food is withdrawn in the mid- to late L4 stage, feeding and colonization on live bacteria were stopped at 46 h post-L1 on plates (Siedel et al. 2011).

- (lines 104-107): In these experiments, synchronized adult N2 worms were used for colonization to provide a genetically homogeneous population of mature hosts, and we allowed worms to colonize in liquid bacteria culture to ensure that all the worms experienced a uniform environment

The robust levels of colonization of the CMC digesting bacteria in Figure 2D relative to the native worm symbionts (Figure S2) is impressive, and makes the implications of the study more interesting.

We agree with the reviewer, and think this information can be utilized for other bacteria-animal interaction systems.

Much more information on the endoglucanase expressing P. Putida is needed. Currently it's just a blurb in the methods. Why was P. Putida chosen as opposed to any other non CMC utilizing bacterium? Why was endoglucanase chosen as opposed to any other carbohydrate active enzyme? What is the ice-nucleation protein and why was it chosen? Were the constructed pET24a plasmids sequence verified prior to or subsequent to transformation (it doesn't sound like it based on the current methods)? Plasmid maps and annotated sequences should be provided.

We have added the following information in lines 632-640: "We used *P. putida* because it is a *Pseudomonas* strain, so it serves as a good negative control strain for our cellulolytic bacteria, *P. cellulosa*. Endoglucanase was chosen for these experiments because it hydrolyzes cellulose by cutting randomly on the cellulose chain. Ice-nucleation protein is a bacterial surface display tag that has been used to demonstrate the translocation of enzymes across the *P. putida* surface (Yang et al., 2008). *P. putida* was transformed with the pET24a-INP-Endoglucanase plasmid, which had been sequenced prior to transformation, and enzyme activity was checked after protein expression. Since endoglucanase only works by being exposed to the substrate cellulose, the activity of the enzyme should come from the surface-displayed enzyme."

No evidence is provided to support the claim that Gentamicin treatment kills bacteria outside, but not inside the worm. The conclusion that the cellulose-derived benefit came from gut, but not external bacteria, in Figure 3B relies on this claim. Thus, the evidence for the claim must be added.

After colonizing the worm with bacteria, we washed worms with M9 worm buffer + 0.1% Triton X-100 to remove most external bacteria (Vega et al. 2017). The use of gentamicin is to prevent bacterial growth by inhibiting protein synthesis. This supports our hypothesis that these cellulose-derived benefits should come from the gut, but not external bacteria. To better explain this point, we changed sentences in lines 181-185 to: "After we washed the worm with 0.1% Triton X-100 to remove most external bacteria (Vega et al., 2017), gentamicin (10 µg/mL) was used to prevent the growth of bacteria outside the worms, ensuring that nutritional benefits would be conferred by resident bacteria in the gut."

The data in Figure 3B do not convincingly support the argument that carbon from microbiome-

processed CMC benefit host metabolism. The larval assay used in Figure 3C is far more convincing. I recommend moving Figure 3B to the SI and giving it only a brief discussion in the main text.

Figure 3B supports our hypothesis that *C. elegans* benefits from the single sugars using direct sugar reporter from the worm. This figure is different from Figure 3C, which uses larval yield as the indicator of the nutrition status of the worm with the help of the cellulolytic bacteria. We believe that inclusion of the fatty acid synthesis reporter data here provides an additional measure that is important for demonstrating the robustness of the conclusion that carbon from microbiome-processed CMC is accessible to the host.

Why does addition of CMC result in more inhibition of Salmonella infection when P. cellulosa and L. plantarum are present? Is Pc degrading CMC so that Lp has more glucose to grow, and produce beneficial effects? This result is interesting, but very empirical at this point, and not satisfying given that the value of the approach in the ability to understand what is happening due to the simplicity of the system.

Yes, our hypothesis is that Pc degraded CMC to glucose, which helps Lp to grow and thus elevate the beneficial effects toward *Salmonella* infection inhibition. To better explain this point, we added the following hypothesis in lines 228-230: “Our hypothesis is that simple sugars released by *P. cellulosa* promote *L. plantarum*’s capability to fight against the pathogen.”

The first 3 sentences of the discussion are more appropriate for the introduction.

We moved the first three sentences of the Discussion to the Introduction section (lines 60-67).

I do not understand the arguments the authors are making in the 3rd paragraph of the Discussion. In particular: "The introduction of multiple functionally equivalent taxa (as with P. cellulosa and B. subtilis) does not necessarily increase the functional capacity of the community if there is exploitation competition between strains; this can reduce the functional redundancy that would otherwise be a desirable outcome of this colonization 20 strategy. However, in our experiments, the use of a facultatively interacting taxon (L. plantarum) allowed us to add a new functional capacity (resilience against pathogen invasion) by capitalizing on the particular properties of this facultative species..."

Our argument was that if bacterial strains in the community have overlapping functions, it does not necessarily increase the overall cellulose hydrolyzing capability of the community. We have made the following changes to the paper to better explain this point. Lines 252–258: “In our experiments, the use of a facultatively interacting taxon (*L. plantarum*) allowed us to add a new functional capacity (resilience against pathogen invasion) by capitalizing on the particular properties of this facultative species. This new function of the community was boosted when we exposed it to CMC, which can be utilized by cellulolytic *P. cellulosa* in the community, demonstrating that distinct bacteria can interact to combat pathogens via cellulose utilization.”

I also don't understand what they are arguing here: "Our results also indicate that nutritional interactions can enhance the performance of the system." Isn't the microbiome-host paradigm a nutritional interaction to a large extent?

We have changed the sentence, as follows: “Our results are also consistent with current thought on the use of “prebiotic” nutritional adjuvants to support the engraftment and function of specific beneficial microbes in the gut.”

The idea of engineering the microbiome to introduce new functionality (final paragraph) is exciting.

Thank you for this comment.

Minor

The details of the Congo Red assay should be moved from the Results to the Materials and Methods.

We moved the Congo Red assay to the Materials and Methods. Additionally, we further modified the main text as follows (lines 86-89): “Additionally, we checked the cellulose degradation capacity for each strain using a Congo Red assay (Houfani et al., 2017; Teather and Wood, 1982). As Congo Red stains glucose polymers, a halo surrounding the colony indicates cellulase activity.”

Table S1 did not render well.

We reworked Table S1.

Numerous supplemental figures are called out incorrectly in the main text (e.g. Figure S4, Figure S5).

We modified the paper to ensure that all supplemental figures are called out correctly.

Lines 159-162: “A high-throughput antibiotic susceptibility screen of the bacterial strains (including *C. elegans* native gut bacteria and the soil bacteria in Table S1) was conducted to determine an appropriate antibiotic cocktail to eliminate these species from the *C. elegans* gut (Fig. S5).”
Lines 162-163: “Clearance by the antibiotic cocktail was tested by imaging worms fed with fluorescently labeled *Pseudomonas putida* (Fig. S6).”
Lines 171-176: “We first validated that *fat-7p*::GFP, which is expressed in the intestinal cells of *C. elegans*, was a reliable reporter for carbon uptake by the host, and determined an optimal glucose concentration of 0.1%-0.2% (w/v) for these assays (Fig. S7).”

Reviewer #3:

This paper claims to "expand host functionality by modifying the gut microbiome" and to "engineer microbiome communities". Unfortunately, it does not convincingly do either. The paper describes two loosely connected observations that potentially could be the start of two rigorous studies. As presented, there is no major advance or novel insight and the paper is mediocre at best. Conclusions that C. elegans uses cellulose broken down by P. cellulosa are not sufficiently supported by the data. In all experiments, worms are colonized with a mixture of P. cellulosa and E. coli. It is possible that the effects observed are indirect, where either bacteria responds to cellulose breakdown products and this in turn affects the worm. Further, the fact that none of the bacteria that are actually found in the wild in the C. elegans gut can metabolize cellulose makes the whole observation less relevant, at least for C. elegans biology. Finally, the paper is overall poorly constructed, the language and descriptions are often vague, there are numerous incorrect references to figures, and, most critically, it lacks rigor.

We appreciate the reviewer's concerns, and have addressed these through the revisions to the text and responses below.

1. In our study, we concluded that *C. elegans* colonized with *P. cellulosa* benefit from CMC utilization by comparing with our negative control when only *E. coli* are present. As shown in Fig. 3B, we used fat-7p::GFP worm to demonstrate that *C. elegans* directly benefit from breakdown of CMC by *P. cellulosa*. It is true that these results do not and cannot distinguish between a direct benefit (where the worm benefits from released carbon directly) and an indirect benefit (where *P. cellulosa* in the gut break down and incorporate carbon from cellulose into their own biomass, and the worm host benefits by digesting *P. cellulosa* biomass in the gut). However, for our purposes, the net effect is the same – the worm host is able to incorporate carbon released from cellulose by the gut-colonizing bacteria, resulting in an increase in available energy (as measured by *fat-7* promoter activity) and increased generation of new biomass (as measured by isotope incorporation and by fecundity).
2. It is true that none of the native worm microbiome isolates in our possession were able to break down cellulose; however, in the broader native worm microbiome, there have been found Bacillales strains that are able to degrade complex polysaccharides including cellulose (Zimmerman et al 2020). It does seem to be true that this function is not extremely common in the native gut microbiome, indicating that *C. elegans* in the wild may have limited ability to utilize cellulose as a nutrient source. <u>However, the purpose of our study was not to recapitulate a native function of the worm microbiome, but to confer a new function by successfully introducing a gut-colonizing microbe that is not native to this host</u>. It makes our approach novel because we can change the animal nutritional phenotype by colonizing bacteria with new functions in the gut. The text has been modified to clarify the context of our results (lines 89-92): "Although investigation of the functional capacity of the *C. elegans* native microbiome indicates that some rare members of this microbiome can degrade cellulose, none of the native *C. elegans* microbiome isolates in this particular set of strains (Fig. 1A) exhibited detectable cellulase activity, which reflects their lack of cellulose processing capability".
3. We went through the manuscript again and have made changes accordingly, to make the presentation clearer and more rigorous.

Other comments:

The title is a massive overstatement of the presented work.

To address the reviewer's point, we altered the title to the more specific: "Enhancing nutritional niche and host defenses by modifying the gut microbiome".

The cartoons are not necessary.

We moved this figure to the last figure of this manuscript (Fig. 4) so it will serve as a summary figure.

Controls are frequently missing.

We double-checked and confirmed that all the experiments have proper controls. We have added all the data points and significance values to the figures.

For many experiments it is not clear how many replicates were done.

To address this point, we added the following information to the revised paper:
Line 309: “All experiments were performed in triplicates.”
Line 323: “All experiments were performed in triplicates.”
Line 341: “All experiments were performed in triplicates.”

Glucose as a carbon source further enhanced colonization density because free sugar monomers are an easier to access carbon source (page 4) - this is interpretation not substantiated by data.

This interpretation is based on our literature summary that bacteria can utilize glucose but not CMC as a carbon source. Thus, CMC needs to be hydrolyzed to glucose monomers first before being utilized. Therefore, CMC itself as a nutrient source without hydrolyzing serves as a negative control and proves our hypothesis that free sugar monomers are an easier to access carbon source. To clarify this point, we changed the relevant text in the paper to following (lines 137-139): “Glucose as a carbon source further enhanced colonization density because free sugar monomers are an easier-to-access carbon source than CMC, which needs to be hydrolyzed to glucose before being utilized.”

There is no evidence that CMC is actually hydrolyzed in the C. elegans gut.

After colonizing the worms with bacteria, we washed the worms with M9 worm buffer + 0.1% Triton X-100 to remove most external bacteria (Vega et al. 2017). Gentamicin was used to prevent bacterial growth by inhibiting protein synthesis. This supports our hypothesis that these cellulose-derived benefits should come from the gut, but not external bacteria. To better explain this point, we changed the relevant text to the following (lines 181-185): “After we washed the worm with 0.1% Triton X-100 to remove most external bacteria (Vega et al., 2017), gentamicin (10 µg/mL) was used to prevent the growth of bacteria outside the worms, ensuring that nutritional benefits would be conferred by resident bacteria in the gut.”

The numbers are very different in figures 2D and 2E: ~700 CFU/worm in 2D, but more than ~3000 in 2E. Also, how are E. coli and P. cellulosa colonies distinguished from each other?

E. coli and *P. cellulosa* can be distinguished by culturing them on different culture medium plates. When we checked on the CFU of *P. cellulosa* in the worm, after feeding bacteria to *C. elegans,* we disrupted the worm and plated them on minimal medium with CMC as the sole carbon source. In that case, *P. cellulosa* will grow but not *E. coli*. For Figures 2D and 2E, we did see some variance of CFU/worm when we did the colonization experiments because of the system complexity. However, the overall range and the trends were reliable, because we have triplicate samples for all the experiments.

They use Pfat-7::GFP as a "reporter of nutritional status" - what does that mean? The results shown in Figure 3B are not convincing. Also, why not just show images, using the sorter makes no sense for this and is not the standard in the field.

The *fat-7p*::GFP reporter, as indicated by the original and cited reference, is a reporter of fatty acid synthesis in the worm. The reporter is known to be induced by glucose (as we confirm in Fig. S7B and Fig. 2B) and can be used to measure active synthesis of fats when carbon is abundant. For clarity, we have modified the following text to motivate the use of this reporter

(lines 172-174): “We first validated that fat-7p::GFP, which is expressed in the intestinal cells of C. elegans (Ma et al., 2015; Nomura et al., 2010), was a reliable reporter for carbon uptake by the host, and determined an optimal glucose concentration of 0.1%-0.2% (w/v) for these assays (Fig. S7).”

Use of the BioSorter allows measurement of fluorescence from this reporter in a larger number of individual worms than is feasible using canonical microscopy and image analysis, giving us greater power to distinguish differences between conditions in our assays. There are numerous papers using *C. elegans* large object sorter data to take observations over populations of worms, and/or presenting detailed protocols for this approach ((Boyd et al., 2010; Fuhrman et al., 2008; Muthaiyan Shanmugam and Subhra Santra, 2016; Twumasi-Boateng et al., 2014)). The addition of microscopy images would add only confirmation of localization of the reporter to the intestine; this has already been described in the original reference, and the localization is not important for our results.

The "reproductive fitness" experiments (normally phrased as "brood size") are not convincing either. On E. coli OP50, worms usually have a brood size of 200 or more. Egg laying dynamics can change depending on nutritional input, so a time course is more useful. Also, there is no reason to do this experiment in 384 well plates, where worms run out of food quickly and larvae are hard to count. Bigger plates (or tubes) with more worms would be more appropriate and is feasible because there are only six conditions tested.

The reviewer is correct that worms on OP50 plates generate much larger numbers of offspring than worms in liquid culture and under nutrient limitation, as used in this assay. Under the conditions used here, we do expect very low fecundity; in the absence of the carbon source, we expect a measurable fraction of the young adult worms used in these experiments to show complete or near-complete reproductive diapause at 48 h, as we observe in Figure 3C. As part of setting up these experiments, we determined that 48 h was an ideal time point for comparison; at 24 h, there were still a large number of zero larval counts across conditions as worms had not all begun laying eggs and early eggs were still in the process of hatching, and dehydration began to become an issue by 72 h. The Methods have been modified to include this information.

We did not find it particularly difficult to count larvae using the protocol described, but acknowledge that this may differ depending on the operator.

Here we chose to measure fecundity in individual worms in liquid media to ensure accuracy of the data. First, as described in the text, it is a peculiarity of the *C. elegans* system that differences in reproductive output based on nutritional availability are maximized when worms are starved at a precise point in the life cycle. We needed to ensure that colonization by live bacteria occurred before that point, and that after that point, worms were provided with only a small amount of inert, non-colonizing bacterial food (here, heat-killed OP50) + the carbon source – hence the use of the 46-h colonization period after L1 plating as described in the main text and Methods. Further, it was desirable to ensure that all worms had uniform access to the inert bacterial food and the carbon source during the fecundity trials. On a purely practical level, this was simpler to accomplish with a transfer to liquid culture.

Second, individual worm measurements of fecundity are difficult to do in standard plating assays due to space and handling time constraints with the large number of individual worms required for an adequate data set. It is well known that both bacterial colonization in the worm gut and fecundity can vary across individual hermaphrodites, even within isogenic, synchronized cultures, and measurement of average phenotypes (including but not limited to fecundity) using

small batches of hermaphrodites often produces data with weird variation structures due to the distribution of rare events. Essentially, this approach takes a linear average of events that are often better described on a log scale; apart from ease of use, it is not clear why this approach should be the standard. By measuring reproductive output in individual worms, we sought to prevent this issue and to describe a representative distribution of individual fecundities, thereby increasing our power to detect differences between groups. This information has been added to the Methods of the revised paper, in the section detailing measurements of fecundity in the worm (lines 578-600).

Reference:

Boyd, W.A., McBride, S.J., Rice, J.R., Snyder, D.W., and Freedman, J.H. (2010). A high-throughput method for assessing chemical toxicity using a Caenorhabditis elegans reproduction assay. Toxicol. Appl. Pharmacol.

Fuhrman, L.E., Shianna, K. V., and Aballay, A. (2008). High-throughput isolation and mapping of C. elegans mutants susceptible to pathogen infection. PLoS One.

Houfani, A.A., Větrovský, T., Baldrian, P., and Benallaoua, S. (2017). Efficient screening of potential cellulases and hemicellulases produced by Bosea sp. FBZP-16 using the combination of enzyme assays and genome analysis. World J. Microbiol. Biotechnol. *33*, 1–6.

Muthaiyan Shanmugam, M., and Subhra Santra, T. (2016). Microfluidic Devices in Advanced Caenorhabditis elegans Research. Molecules.

Teather, R.M., and Wood, P.J. (1982). Use of Congo red-polysaccharide interactions in enumeration and characterization of cellulolytic bacteria from the bovine rumen. Appl. Environ. Microbiol. *43*, 777–780.

Twumasi-Boateng, K., Berg, M., and Shapira, M. (2014). Automated separation of C. elegans variably colonized by a bacterial pathogen. J. Vis. Exp.

Yang, C., Cai, N., Dong, M., Jiang, H., Li, J., Qiao, C., Mulchandani, A., and Chen, W. (2008). Surface Display of MPH on Pseudomonas putida JS444 Using Ice Nucleation Protein and Its Application in Detoxification of Organophosphate, Biotechnol. Bioeng., *99*, 30-37.

Thank you for sending us your revised manuscript. We have now heard back from the reviewer who was asked to evaluate your study. As you will see below, they are satisfied with the modifications made and are supportive of publication. Before we formally accept the study for publication, we would also ask you to address some remaining editorial issues listed below:

Reviewer #2:

The authors have addressed most of my concerns.

We are submitting the revised version of our manuscript, "Enhancing nutritional niche and host defenses by modifying the gut microbiome," by Q. Sun and N.M. Vega et al. We appreciate the effort and time from you and the reviewers. A few minor changes were made in the text.

Our responses to the reviewers' comments are noted in blue below.

Before we formally accept the study for publication, we would also ask you to address some remaining editorial issues listed below:
- Our data editors have noticed some unclear or missing information in the figure legends, please see the attached .doc file. Please make all requested text changes using the attached file and *keeping the "track changes" mode* so that we can easily access the edits made.
Thank you for noticing the missing information in the figure legends. We have made all requested changes using the "track changes" mode.

- Please provide individual production quality figure files for the main and EV Figures (one file per figure).
We have provided all the individual production-quality figures with this submission.

- The figures (and the Appendix Figures) need to be removed from the main manuscript text. The figure legends for the main figures should be provided all together at the end of the main text, before the References.
We have removed all the figures from the main manuscript text, as well as the supplementary information, and put all the figure legends together.

- Please make sure all Appendix figures are called out in the main text. Currently, callouts to Appendix Figures S3, S4, S8 are missing.
Thank you for noticing that. We have added references to the Appendix Figures S3 and S4 on page 4, and to S8 on page 6.

- Please provide a "standfirst text" summarizing the study in one or two sentences (approximately 250 characters), three to four "bullet points" highlighting the main findings and a "synopsis image" (550px width and max 400px height, jpeg format) to highlight the paper on our homepage.
We have provided these descriptions here.

We developed a microbe-host interaction model by colonizing *Caenorhabditis elegans* with functional bacteria to digest long-chain cellulose. Direct benefits include increased host larval yield and protection of other gut species against pathogens.

- Heterologous bacteria such as *Pseudomonas cellulosa* colonizing the *C. elegans* gut can help the worm to digest cellulose, an otherwise indigestible substrate. Carbon derived from the breakdown of cellulose then provides nutrition for the host.
- Cellulolytic bacteria in the worm gut can also support other bacterial species with specialized roles: *Lactobacillus* protected the worms against Salmonella infection, and interspecies synergy between *Pseudomonas cellulosa* and *Lactobacillus plantarum* conferred benefit to the host.
- Engineered microbiome communities may provide host organisms with novel functions, such as the ability to use complex nutrient sources and to fight against pathogen infection.
- *C. elegans* colonized with bacteria provides a model system for the study of microbiome-host interactions.

We have included the synopsis image. Please let us know if you have any questions.

- All Materials and Methods should be described in the main text and NOT in the Appendix.
We have put all materials and methods in the main text.

- Appendix Table S1 looks truncated in the PDF, please check.
We have fixed Table S1 as requested and attached the new Table S1 in this submission.

- The data availability section should be moved to the main text, together with the Materials and Methods. Please provide all relevant data as EV Datasets and amend the Data Availability statement accordingly. Our editorial policy does not allow "data available upon request".
We have moved the data availability section to the main text together with the Materials and Methods. We have also removed the sentence "data available upon request".

2nd Revision - Editorial Decision 14th Oct 2022

Thank you again for sending us your revised manuscript. We are now satisfied with the modifications made and I am pleased to inform you that your paper has been accepted for publication.

EMBO PRESS

YOU MUST COMPLETE ALL CELLS WITH A PINK BACKGROUND ↓

PLEASE NOTE THAT THIS CHECKLIST WILL BE PUBLISHED ALONGSIDE YOUR PAPER

Timothy K. Lu
Molecular Systems Biology
MSB-20-9933

Reporting Checklist For Life Sciences Articles (Rev. June 2017)

This checklist is used to ensure good reporting standards and to improve the reproducibility of published results. These guidelines are consistent with the Principles and Guidelines for Reporting Preclinical Research issued by the NIH in 2014. Please follow the journal's authorship guidelines in preparing your manuscript.

A- Figures
1. Data
The data shown in figures should satisfy the following conditions:

- the data were obtained and processed according to the field's best practice and are presented to reflect the results of the experiments in an accurate and unbiased manner.
- figure panels include only data points, measurements or observations that can be compared to each other in a scientifically meaningful way.
- graphs include clearly labeled error bars for independent experiments and sample sizes. Unless justified, error bars should not be shown for technical replicates.
- if n< 5, the individual data points from each experiment should be plotted and any statistical test employed should be justified
- Source Data should be included to report the data underlying graphs. Please follow the guidelines set out in the author ship guidelines on Data Presentation.

2. Captions

Each figure caption should contain the following information, for each panel where they are relevant:

- a specification of the experimental system investigated (eg cell line, species name).
- the assay(s) and method(s) used to carry out the reported observations and measurements
- an explicit mention of the biological and chemical entity(ies) that are being measured.
- an explicit mention of the biological and chemical entity(ies) that are altered/varied/perturbed in a controlled manner.
- the exact sample size (n) for each experimental group/condition, given as a number, not a range;
- a description of the sample collection allowing the reader to understand whether the samples represent technical or biological replicates (including how many animals, litters, cultures, etc.).
- a statement of how many times the experiment shown was independently replicated in the laboratory.
- definitions of statistical methods and measures:
  - common tests, such as t-test (please specify whether paired vs. unpaired), simple χ2 tests, Wilcoxon and Mann-Whitney tests, can be unambiguously identified by name only, but more complex techniques should be described in the methods section;
  - are tests one-sided or two-sided?
  - are there adjustments for multiple comparisons?
  - exact statistical test results, e.g., P values = x but not P values < x;
  - definition of 'center values' as median or average;
  - definition of error bars as s.d. or s.e.m.

Any descriptions too long for the figure legend should be included in the methods section and/or with the source data.

**In the pink boxes below, please ensure that the answers to the following questions are reported in the manuscript itself. Every question should be answered. If the question is not relevant to your research, please write NA (non applicable).
We encourage you to include a specific subsection in the methods section for statistics, reagents, animal models and human subjects.**

USEFUL LINKS FOR COMPLETING THIS FORM

http://www.antibodypedia.com	Antibodypedia
http://1degreebio.org	1DegreeBio
http://www.equator-network.org/reporting-guidelines/improving-bioscience-research-repo	ARRIVE Guidelines
http://grants.nih.gov/grants/olaw/olaw.htm	NIH Guidelines in animal use
http://www.mrc.ac.uk/Ourresearch/Ethicsresearchguidance/Useofanimals/index.htm	MRC Guidelines on animal use
http://ClinicalTrials.gov	Clinical Trial registration
http://www.consort-statement.org	CONSORT Flow Diagram
http://www.consort-statement.org/checklists/view/32-consort/66-title	CONSORT Check List
http://www.equator-network.org/reporting-guidelines/reporting-recommendations-for-tun	REMARK Reporting Guidelines (marker prognostic studies)
http://datadryad.org	Dryad
http://figshare.com	Figshare
http://www.ncbi.nlm.nih.gov/gap	dbGAP
http://www.ebi.ac.uk/ega	EGA
http://biomodels.net/	Biomodels Database
http://biomodels.net/miriam/	MIRIAM Guidelines
http://jjj.biochem.sun.ac.za	JWS Online
http://oba.od.nih.gov/biosecurity/biosecurity_documents.html	Biosecurity Documents from NIH
http://www.selectagents.gov/	List of Select Agents

B- Statistics and general methods

Please fill out these boxes ↓ (Do not worry if you cannot see all your text once you press return)

1.a. How was the sample size chosen to ensure adequate power to detect a pre-specified effect size?	Data was analyzed from 3 replicates (sample size) based on standard sample size used for statistical calculation in biological experiments
1.b. For animal studies, include a statement about sample size estimate even if no statistical methods were used.	All worm experiments were repeated 3 times.
2. Describe inclusion/exclusion criteria if samples or animals were excluded from the analysis. Were the criteria pre-established?	No data were excluded
3. Were any steps taken to minimize the effects of subjective bias when allocating animals/samples to treatment (e.g. randomization procedure)? If yes, please describe.	Worms from each conditions that were imaged/characterized were randomly selected.
For animal studies, include a statement about randomization even if no randomization was used.	Worms from each conditions that were imaged/characterized were randomly selected.
4.a. Were any steps taken to minimize the effects of subjective bias during group allocation or/and when assessing results (e.g. blinding of the investigator)? If yes please describe.	No blinding was used in these experiments. The experimental protocol was identical for worms at different experimental settings. Data analysis was automated and required no input from investigators.
4.b. For animal studies, include a statement about blinding even if no blinding was done	No blindig was done.
5. For every figure, are statistical tests justified as appropriate?	Yes. All statistical tests were justified.
Do the data meet the assumptions of the tests (e.g., normal distribution)? Describe any methods used to assess it.	Normal distribution was assumed for all the experiments. Student's t-test was used to test the hypothesis and P value was used and labeled in the figure to validate our hypothesis.

Is there an estimate of variation within each group of data?	No.
Is the variance similar between the groups that are being statistically compared?	Yes. We used student's t-test throughout the figures.

C- Reagents

6. To show that antibodies were profiled for use in the system under study (assay and species), provide a citation, catalog number and/or clone number, supplementary information or reference to an antibody validation profile. e.g., Antibodypedia (see link list at top right), 1DegreeBio (see link list at top right).	N/A
7. Identify the source of cell lines and report if they were recently authenticated (e.g., by STR profiling) and tested for mycoplasma contamination.	N/A

* for all hyperlinks, please see the table at the top right of the document

D- Animal Models

8. Report species, strain, gender, age of animals and genetic modification status where applicable. Please detail housing and husbandry conditions and the source of animals.	C. elegans were utilized. The species, strain, age of animals and genetic modification status were detailed in the manuscript.
9. For experiments involving live vertebrates, include a statement of compliance with ethical regulations and identify the committee(s) approving the experiments.	N/A
10. We recommend consulting the ARRIVE guidelines (see link list at top right) (PLoS Biol. 8(6), e1000412, 2010) to ensure that other relevant aspects of animal studies are adequately reported. See author guidelines, under 'Reporting Guidelines'. See also: NIH (see link list at top right) and MRC (see link list at top right) recommendations. Please confirm compliance.	N/A

E- Human Subjects

11. Identify the committee(s) approving the study protocol.	N/A
12. Include a statement confirming that informed consent was obtained from all subjects and that the experiments conformed to the principles set out in the WMA Declaration of Helsinki and the Department of Health and Human Services Belmont Report.	N/A
13. For publication of patient photos, include a statement confirming that consent to publish was obtained.	N/A
14. Report any restrictions on the availability (and/or on the use) of human data or samples.	N/A
15. Report the clinical trial registration number (at ClinicalTrials.gov or equivalent), where applicable.	N/A
16. For phase II and III randomized controlled trials, please refer to the CONSORT flow diagram (see link list at top right) and submit the CONSORT checklist (see link list at top right) with your submission. See author guidelines, under 'Reporting Guidelines'. Please confirm you have submitted this list.	N/A
17. For tumor marker prognostic studies, we recommend that you follow the REMARK reporting guidelines (see link list at top right). See author guidelines, under 'Reporting Guidelines'. Please confirm you have followed these guidelines.	N/A

F- Data Accessibility

18: Provide a "Data Availability" section at the end of the Materials & Methods, listing the accession codes for data generated in this study and deposited in a public database (e.g. RNA-Seq data: Gene Expression Omnibus GSE39462, Proteomics data: PRIDE PXD000208 etc.) Please refer to our author guidelines for 'Data Deposition'.<br><br>Data deposition in a public repository is mandatory for:<br>a. Protein, DNA and RNA sequences<br>b. Macromolecular structures<br>c. Crystallographic data for small molecules<br>d. Functional genomics data<br>e. Proteomics and molecular interactions	Data supporting the findings of this study are available within the papr and its Supplementary Information files. Source data are provided within this paper. The datasets generated and analyzed during the current study are available from the corresponding author upon request. A reporting summary for this article is availabl as a Supplementary Information file.
19. Deposition is strongly recommended for any datasets that are central and integral to the study; please consider the journal's data policy. If no structured public repository exists for a given data type, we encourage the provision of datasets in the manuscript as a Supplementary Document (see author guidelines under 'Expanded View' or in unstructured repositories such as Dryad (see link list at top right) or Figshare (see link list at top right).	N/A
20. Access to human clinical and genomic datasets should be provided with as few restrictions as possible while respecting ethical obligations to the patients and relevant medical and legal issues. If practically possible and compatible with the individual consent agreement used in the study, such data should be deposited in one of the major public access-controlled repositories such as dbGAP (see link list at top right) or EGA (see link list at top right).	N/A
21. Computational models that are central and integral to a study should be shared without restrictions and provided in a machine-readable form. The relevant accession numbers or links should be provided. When possible, standardized format (SBML, CellML) should be used instead of scripts (e.g. MATLAB). Authors are strongly encouraged to follow the MIRIAM guidelines (see link list at top right) and deposit their model in a public database such as Biomodels (see link list at top right) or JWS Online (see link list at top right). If computer source code is provided with the paper, it should be deposited in a public repository or included in supplementary information.	N/A

G- Dual use research of concern

22. Could your study fall under dual use research restrictions? Please check biosecurity documents (see link list at top right) and list of select agents and toxins (APHIS/CDC) (see link list at top right). According to our biosecurity guidelines, provide a statement only if it could.	
